# Genomic map of the functionally extinct northern white rhinoceros (*Ceratotherium simum cottoni*)

**DOI:** 10.1073/pnas.2401207122

**Published:** 2025-05-13

**Authors:** Gaojianyong Wang, Marisa L. Korody, Björn Brändl, Camilo Jose Hernandez-Toro, Christian Rohrandt, Karl Hong, Andy Wing Chun Pang, Joyce Lee, Giovanna Migliorelli, Mario Stanke, Sarah M. Ford, Iris Pollmann, Marlys L. Houck, Harris A. Lewin, Teri L. Lear, Oliver A. Ryder, Alexander Meissner, Jeanne F. Loring, Franz-Josef Müller

**Affiliations:** ^a^Department of Genome Regulation, Max Planck Institute for Molecular Genetics, Berlin 14195, Germany; ^b^Department of Psychiatry and Psychotherapy, Christian-Albrechts Universität, Kiel 24105, Germany; ^c^Zentrum für Integrative Psychiatrie, University Hospital Schleswig-Holstein, Kiel 24105, Germany; ^d^San Diego Zoo Wildlife Alliance, Escondido, CA, 92027; ^e^Institute for Communications Technologies and Embedded Systems, Kiel University of Applied Sciences, Kiel 24149, Germany; ^f^Bionano Genomics Inc, San Diego CA, 92121; ^g^Institute of Mathematics and Computer Science, and Center for Functional Genomics of Microbes, University of Greifswald, Greifswald 17489, Germany; ^h^Department of Ecology and Evolutionary Biology, University of California, Santa Cruz, CA 95060; ^i^The Genome Center, University of California, Davis, CA 95616; ^j^Department of Evolution and Ecology, University of California, Davis, CA 95616; ^k^John Muir Institute for the Environment, University of California, Davis, CA 95616; ^l^Gluck Equine Research Center, Department of Veterinary Science, University of Kentucky, Lexington, KY 40546; ^m^Scripps Research, La Jolla, CA 92037

**Keywords:** northern white rhinoceros, southern white rhinoceros, genome assembly, genome integrity, iPSC

## Abstract

The northern white rhinoceros (NWR; *Ceratotherium simum cottoni*) is functionally extinct, with only two nonreproductive females alive. Efforts to rescue the NWR from its inevitable demise have inspired the exploration of unconventional conservation methods, including the development of induced pluripotent stem cells (iPSCs) for the in vitro generation of artificial gametes. The integrity of iPSC genomes is critical for in vitro gametogenesis to be used for assisted reproductive technologies using NWR iPSCs. We generated a chromosome-level NWR reference genome that meets or exceeds the metrics proposed by the Vertebrate Genome Project, using complementary sequencing and mapping methods. The genome represents 40 autosomes, an X and a partially resolved Y chromosome, and the mitochondrial genome. Using comparative FISH mapping, we confirmed a general gene order conservation between the NWR and horse genomes. We aligned the NWR genome with that of the southern white rhinoceros (SWR; *Ceratotherium simum simum*), a population that has been physically separated from the NWR for tens of thousands of years, and we found that the two subspecies are very similar on the chromosome level. Comparing long-read data from NWR iPSC lines and the fibroblast cultures used for reprogramming, we identified copy number variations that were likely to have been introduced during in vitro iPSC expansion. The NWR reference genome allows for efficient, rapid, and accurate assessment of the genomic integrity of iPSC lines to direct their differentiation. This will assist in strategies to rescue the NWR through extraordinary measures like cloning and the generation of embryos from iPSC-derived gametes.

## Significance

The northern white rhinoceros (NWR; *Ceratotherium simum cottoni*) is functionally extinct, with only two nonreproductive females remaining alive. Extraordinary measures are underway to rescue this species, including using a collection of NWR induced pluripotent stem cells (iPSCs) to generate gametes for assisted reproduction technologies. Because of the critical importance of genomic integrity in germ cells used for reproduction, these approaches require extensive genomic analyses to exclude aberrations that are acquired during culture of iPSCs. In order to support those efforts, we have generated a chromosome-level genome assembly of northern white rhinoceros and used this reference genome to evaluate the genomic integrity of iPSCs cultured for the generation of artificial gametes.

The northern white rhinoceros (NWR; *Ceratotherium simum cottoni*), with only two nonreproductive females currently alive, is functionally extinct due to human activities including poaching, civil war, and habitat loss and fragmentation (1–3). The development of assisted reproductive technologies such as fertilization of harvested eggs with intracytoplasmic sperm injection (4), somatic cell nuclear transfer (SCNT), and generation of artificial gametes from induced pluripotent stem cells (iPSCs) may provide ways to save the NWR from extinction (5). As part of an international plan to reestablish the NWR (6), we have reported generation of iPSCs from nine of the twelve NWR individuals and two southern white rhinoceros (SWR; *Ceratotherium simum simum*) whose fibroblasts are cryopreserved in the Frozen Zoo^®^ (7, 8). Based on genomic data from the NWRs, the nine iPSC lines are believed to encompass sufficient genomic diversity to reestablish a viable animal population (9–11). Mouse pluripotent stem cells have been differentiated into oocytes, which have been used to produce viable offspring (12–14). If the same can be achieved for NWR, it may be possible to generate embryos for implantation into SWR surrogate mothers. In an important step toward generation of artificial gametes, it was recently reported that primordial germ cells, the embryonic precursors of gametes, have been derived from NWR iPSCs (15).

The well-annotated reference genomes of the human and mouse have been foundational for the iPSC field, providing genomic tools for research and for quality control in development of stem cell–based human therapies. A critical step in the derivation of iPSC lines is a comprehensive assessment of genomic integrity (16) because pluripotent stem cells often acquire multiple types of genomic aberrations during expansion in vitro (17–19) that may be deleterious to gametes, embryos, or offspring. Mutations and copy number variations (CNVs: deletions, duplications, and loss of heterozygosity) are often lethal during embryonic development or cause severe defects in both mice and humans (20, 21). The lack of detailed genomic information has hampered development of genetic rescue technologies for endangered species.

Combining multiple tools, including linked reads, HiC mapping, third-generation sequencing methods, and optical genome mapping (OGM; Bionano Genomics), along with major advances in computational approaches, has greatly improved de novo assembly of genomes from nonmodel species. These technologies have helped reduce gaps in genome sequences and capture complex regions that were previously difficult to assemble. The Vertebrate Genomes Project (VGP) has reported best practices, workflows, and results for generating high-quality, near-gapless reference genomes using these methods (22).

Here, we report the genome assembly of a male NWR using long-read, short-read, and OGM technologies. The resulting 2.5 GB genome assembly is highly contiguous. It contains 40 partially haplo-phased, chromosome-level autosomes, the X chromosome, and a partially resolved Y chromosome, with several chromosomes potentially achieving complete telomere-to-telomere contiguity, including the X chromosome and autosomal scaffolds 1, 11, and 23. The overall quality of the genome reaches or exceeds the metrics proposed by the VGP (22). Additionally, we report the long-read assembly of the NWR mitochondrial genome. The generated high-quality NWR reference genome confirms gene order conservation between NWR and horse. In addition, based on FISH (fluorescence in situ hybridization) and OGM from three SWR individuals, we provide evidence for a consistent chromosomal structure shared between the NWR and SWR populations that have been geographically separated for tens of thousands of years (9, 23). The results of this report provide essential resources for unconventional conservation methods.

## Results

### Assembly of the NWR Reference Genome.

**Fig. 1.**
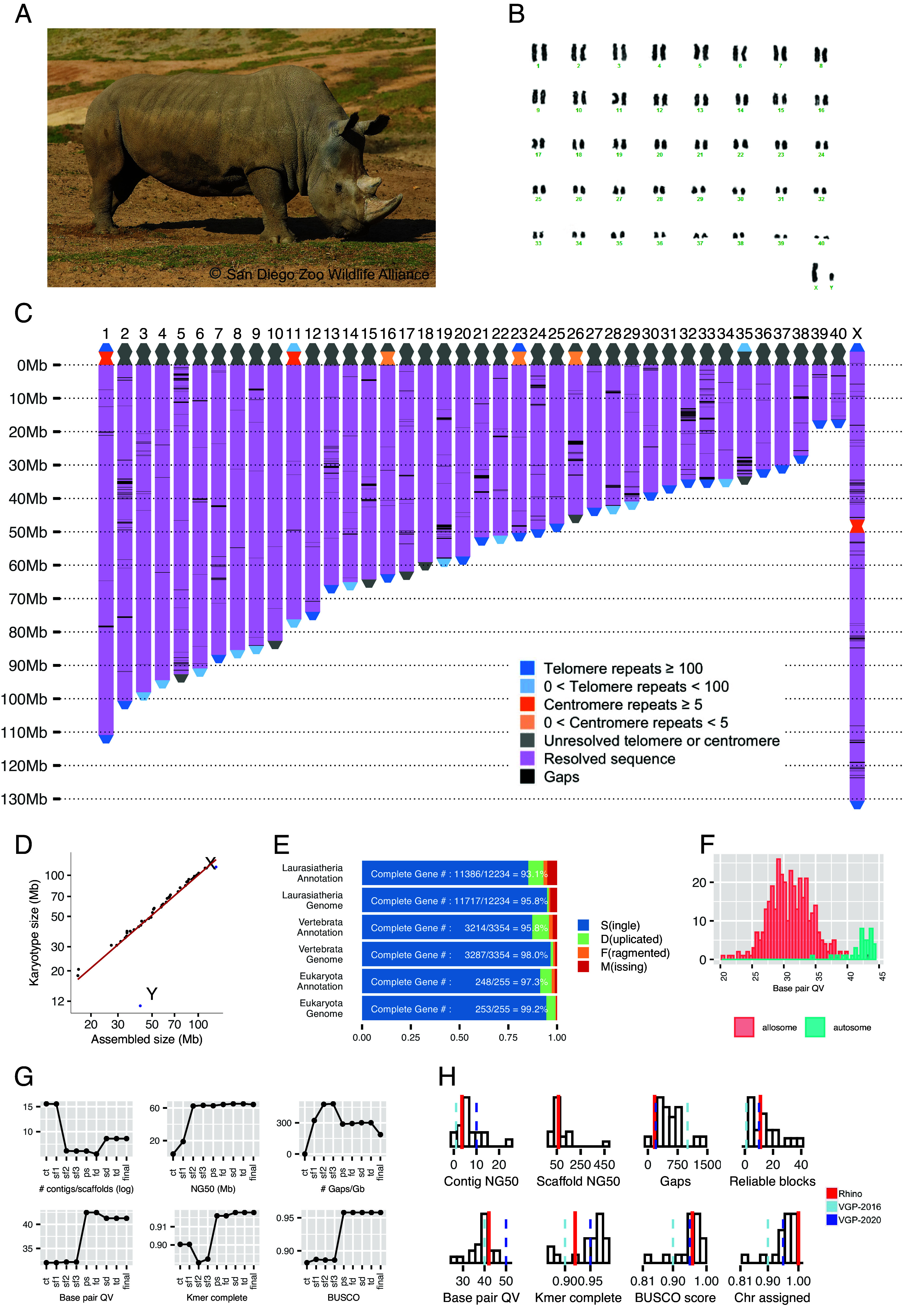
NWR genome assignment into chromosome-equivalent scaffolds and quality metrics. (*A*) Angalifu: 01 April 1972 (est.) - 14 December 2014 (photo credit: San Diego Zoo Wildlife Alliance). (*B*) Giemsa-stained karyotype of Angalifu NWR 9947-c501 iPSCs, indicating a 2n = 82 karyotype. (*C*) Visualization of the assembled NWR reference genome indicating resolved regions and remaining gaps. (*D*) The size of the assembled chromosome-level scaffolds compared to the estimated chromosome sizes derived from the karyotype. (*E*) Benchmark Universal Single Copy Ortholog (BUSCO) scores for the genome assembly and annotation using three datasets: eukaryota (255 genes), Vertebrata (3,354 genes), and Laurasiatheria (12,234 genes). (*F*) The improvements in genome quality for each metric throughout the assembly process. (*G*) The histograms of autosomes and allosomes base pair QV. (*H*) The quality of the NWR reference genome compared to the metrics proposed by the Vertebrate Genome Project, i.e., VGP-2016 and VGP-2020. The histograms represent the qualities of the 16 genomes generated by VGP (22).

We obtained fibroblasts and an iPSC line (NWR 9947-c501) from a male NWR individual (“Angalifu”, * April 1972 (est) - † December 14, 2014, laboratory number KB9947, Fig. 1*A*) (8). The iPSCs were generated from cryopreserved fibroblasts, the only available source of DNA from this individual. The fibroblasts were collected on October 16, 1997, and cultured and cryopreserved for banking in the Frozen Zoo^®^. The karyotyped NWR cells show 40 pairs of autosomes and one pair of allosomes (Fig. 1*B*), consistent with published data (24). We prepared, extracted, and deeply sequenced high-molecular-weight DNA from these cells using four technologies: Hi-C chromatin conformation capture with an average of 40X coverage (fibroblasts at passage 6, P6), 10X Genomics linked reads (10XG, fibroblasts at P6) with an average of 80X coverage, OGM (OGM; Bionano Genomics) with an average of 400X coverage (fibroblasts at P8), and Oxford Nanopore Technologies (ONT) long reads with an average of 75X coverage (fibroblast at P12 and iPSC at P40, *Materials and Methods*).

#### First draft assembly.

##### Autosome assembly.

We followed an automated and manual assembly strategy similar to the workflow recently proposed by the VGP (22), using ONT long reads instead of PacBio continuous long reads, and customized the pipelines for allosome and MT assembly (*SI Appendix*, Fig. S1). Contiguous sequences (contigs) were first assembled from ONT long reads using the Shasta assembler (25), followed by three sequential rounds of scaffolding using 10XG linked reads, OGM, and Hi-C sequencing data from the same individual (*SI Appendix*). The scaffolds were then polished using Racon (26) and Pilon (27) (*SI Appendix*, Fig. S1*A*). The first draft NWR genome assembly (fd) was generated after manual curation of the polished scaffolds (*SI Appendix*, Fig. S2 *A* and *B*) using Juicebox (28).

##### Allosome assembly.

One assembled chromosome-level scaffold in the first draft had a mean coverage (ONT long reads) of 38X (*SI Appendix*, Fig. S2*C*), which is approximately half the 75X coverage of the other scaffolds and corresponds to the X chromosome because the sequenced NWR individual Angalifu was male. We found that some regions of the X chromosome in the first draft contained more coverage than expected (*SI Appendix*, Fig. S2*C*). Because of the similarities between the X and Y in the pseudoautosomal region (PAR), the repetitive sequences found at the boundaries of the PAR, and other repetitive sequences (such as telomeres and centromeres), the *first draft* contained a hybrid scaffold containing X and Y sequences.

#### Second draft assembly.

Consequently, we reassembled sequences corresponding to the Y chromosome using a custom pipeline and manual curation (*SI Appendix*, Figs. S1*B* and S2*E*) by selecting similar sequences of chromosomes X and Y. Briefly, the reassembled chromosome Y scaffolds were aligned back to the hybrid allosome scaffold in the first draft. The chromosome Y scaffolds that appeared to be misassembled into this hybrid allosome scaffold were removed from the hybrid allosome based on the coverage profile. This resulted in the generation of a trimmed X chromosome (130.6 Mbps in total, updated coverage profile in *SI Appendix*, Fig. S2*D*), a partially resolved Y chromosome (155 scaffolds, 11.1 Mbps in total), and 194 unlocalized contigs. The reassembled chromosome-level X scaffold and Y scaffolds, together with the previous 40 autosome scaffolds, were referred to as the *second draft* NWR genome assembly.

#### Third draft assembly.

##### Mitochondrial Genome.

We employed a long-read bait strategy to exclude nuclear mitochondrial DNA segments (NUMTs) and to assemble the NWR mitochondrial genome (MT). We used the mitochondrial genome of a closely related species, the domestic horse [*Equus caballus*, EquCab3.0, GenBank assembly accession: GCA_002863925.1 (29)], as a reference. The reads with aligned size larger than 1 kbp were selected to construct a primary mitochondrial genome of 16,715 bps (*SI Appendix*, Fig. S1*C*). Subsequently, the MT was curated and added to the *second draft*, resulting in the *third draft* (td) NWR genome assembly. To ensure that the assembly consisted of mitochondrial-originating reads, we first used a k-mer mapping strategy to identify nuclear-mitochondrial DNA segments (NUMTs); the majority were less than 100 bp. The largest three detected NUMTs were 2,556, 2,151, and 1,171 bps long (*SI Appendix*, Fig. S3*A*). In comparison, the bait strategy selected 6,040 reads with an N50 (shortest read that covers half of the assembly) of 14.7 kbp. We then used ONT sequencing to compare the methylation profiles of MT and the three NUMTs. We found that, as previously described in humans (30, 31), mitochondria in NWR carry significantly less CpG methylation than that observed in NUMTs (*SI Appendix*, Fig. S3 *B*–*E*). Therefore, nanopore reads significantly longer than the largest NUMTs with no detectable CpG methylation were used to generate the MT.

##### Curation.

We curated the third draft to close 307 small gaps (*SI Appendix*, Fig. S2*F*) using ONT reads. We called the resulting genome assembly CerSimCot1.0 (GenBank assembly accession: GCA_021442165.1). It contains a complete mitochondrial genome as well as the chromosomal-level scaffolds (Fig. 1*C*). Based on their sizes, we then named the autosomal scaffolds in CerSimCot1.0 “assembled chromosomes” CHR 1 to 40. The X chromosome scaffold in CerSimCot1.0 is named as CHR X and the remaining Y chromosome scaffolds are named CHR Y scaffold 1 to 155. We identified a 228 bps NWR-specific centromeric repeat sequence from short reads (32) (*SI Appendix*). Using this 228 bps sequence as well as the telomeric repeat sequence (TTAGGG), we found that CerSimCot1.0 CHR 1, 11, 23, and X were assembled telomere to telomere with 10, 4, 6, and 64 gaps, respectively, and CHR 16 was assembled centromere to telomere with 7 gaps remaining (Fig. 1*C*).

### Quality of the NWR Reference Genome.

The CerSimCot1.0 genome assembly meets or exceeds the metrics proposed by the VGP (22) (Fig. 1*H* and *SI Appendix*, Table S1). The assembly is 2.50 Gbps with a contig NG50 (the length of the scaffold at which 50% of the genome length is covered) of 3.6 Mbps and a chromosome-level scaffold size that is within 8% of the estimated genome size of 2.69 Gbps based on k-mer analysis (*SI Appendix*). In each step of the genome assembly pipeline, there was an improvement in at least one of the VGP genome quality metrics (Fig. 1*F*). Overall, the CerSimCot1.0 genome assembly has reliable blocks of 9.4 Mbps, phased blocks of 2.0 Mbps, a base pair quality value (QV) of 41.3, and k-mer completeness of 91.8% (*SI Appendix*, Table S1). Due to the complexity and larger extent of repetitive regions within the allosomes, particularly in the Y chromosome (33), the assembly qualities of CHR X and Y (QV range: 23 to 39; Fig. 1*H*) are lower than those of the CHR 1 to 40 (QV range: 34 to 44; Fig. 1*F*). In total, 427 gaps (approximately 160 gaps/Gbps) remain in the final genome assembly (*SI Appendix*, Table S1). Genome completeness was assessed using BUSCO (34) for three datasets: Eukaryota - 99.2% complete; Vertebrata - 98.0% complete; and Laurasiatheria - 95.8% complete (Fig. 1*E* and *SI Appendix*, Table S1).

### Annotation of the NWR Reference Genome.

To annotate the genome, we first masked the CerSimCot1.0 genome assembly (*SI Appendix*), identifying approximately 33.22% of the genome as repetitive or low complexity, including 3.15% in short interspersed nuclear elements, 20.94% in long interspersed nuclear elements, 5.83% in long terminal repeats, and 3.17% in DNA repeat elements (TcMar-Tigger and hAT-Charlie transposons). Genome annotation and functional annotation were then performed using BRAKER3 (35) (*SI Appendix*) with a combination of RNAseq data from multiple tissues and cell types of NWR and SWR (*SI Appendix*, Table S2) and five protein datasets (*SI Appendix*, Table S3). The BUSCO scores for the resulting genome annotation based on three datasets are Eukaryota - 97.6% complete; Vertebrata - 96.0% complete; Laurasiatheria (a group of placental mammals) - 92.0% complete (Fig. 1*E*).

### Chromosome and Gene Order Conservation.

**Fig. 2.**
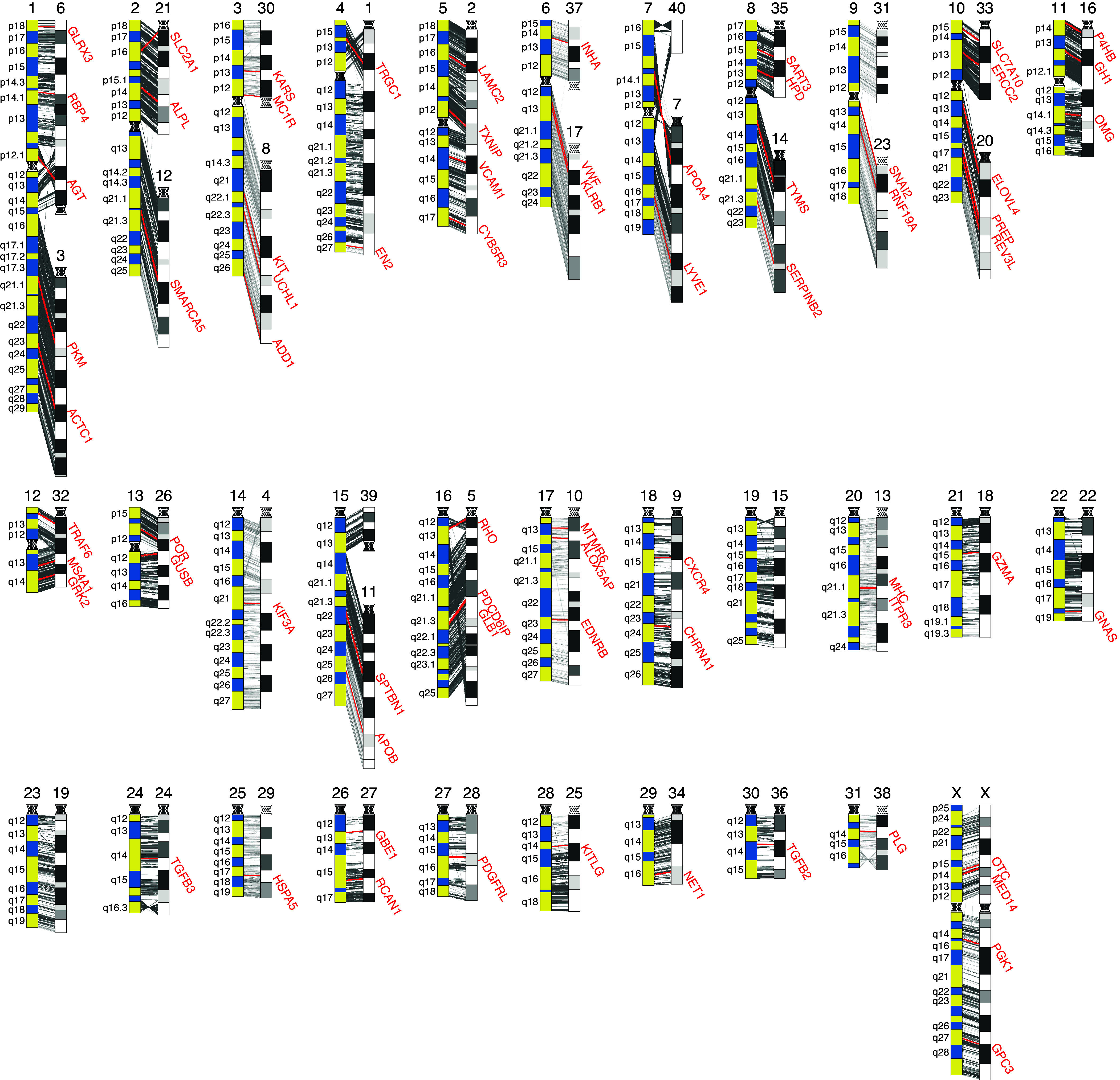
Gene order visualization between horse and NWR genomes represented at the chromosome level as G-banded karyotype ideograms. Gray lines connecting horse ideogram (yellow and blue G-banding) with corresponding NWR ideogram (grayscale G-banding) represent gene orientation between the horse (EquCab3.0) and the NWR genome. Centromeres are shown as dotted regions. Genes/regions with FISH experiments supporting the mapping locations are highlighted in red (*SI Appendix*, Fig. S4 *C*–*E*).

The chromosomal-level scaffolds CHR 1 to 40 and CHR X agree well with the estimated chromosome sizes obtained from the karyotypes (Fig. 1*D*). To orient our scaffolds to the respective chromosomes, we used a combination of G-banded karyotypes and cross-species fluorescent in situ hybridization (FISH) mapping. Cross-species chromosome FISH painting experiments (36, 37) have reported a strong conservation of chromosome segments across the odd-toed ungulates (Order: *Perissodactyla*) despite a range of karyotypes (2n = 32 – 2n = 84) (38). Comparing G-banded karyotypes across rhinoceros species (greater one-horned: 2n = 82, northern white: 2n = 82, southern white: 2n = 82, eastern black: 2n = 84, southern black: 2n = 84, and Sumatran: 2n = 82) identified a conserved banding pattern for all 2n = 82 rhinoceros species (Fig. 2). Mapping 71 domestic horse bacterial artificial chromosomes (BACs) previously mapped in equids (39–41) to these rhinoceros species (*SI Appendix*, Fig. S4*B*) confirmed the conserved G-banding pattern and provided gene anchors for 35 of 40 scaffolds and the X, with a high conservation of mapping orientation and chromosome arm conservation between horse and rhinoceros, which agrees with the previous painting experiments.

To assess conservation of genomes, we compared the domestic horse genome [*Equus caballus*, EquCab3.0, GenBank assembly accession: GCA_002863925.1 (29)] to the CerSimCot1.0 genome assembly. We aligned all annotated genes in EquCab3.0 to CerSimCot1.0 (*SI Appendix*) and observed a strong conservation of the gene order between horse and NWR (Fig. 2 and *SI Appendix*, Fig. S4*A*). We assumed centromere conservation between horse and rhinoceros for chromosomes 18 and 19, which was further confirmed by identifying resolved telomere sequences. We therefore oriented chromosomes 31 and 39 using resolved telomere sequences. Scaffold 40 orientation could not be confirmed with FISH or sequencing data and is therefore left blank in Fig. 2. Chromosome arms and/or whole chromosomes are relatively conserved between the two species despite divergence of 52 to 58 My (37, 42). The reference for the G-banding of the horse ideograms was the standard for the horse as defined by the International System for Cytogenetic Nomenclature of the Domestic Horse (43). The rhinoceros ideogram (*SI Appendix*, Fig. S4*F*) was first presented at the Plant and Animal Genome Conference XIII (44). Our first alignment with EquCab3.0 showed large rearrangements on chromosomes 1, 5, 6, 21, and 35 (*SI Appendix*, Fig. S5). We closely examined the FISH data (*SI Appendix*, Tables S4 and S5) for gene order/orientation and determined that these were assembly errors in regions containing gaps. We manually reoriented regions between the gaps to resolve the gene order errors. Additionally, we performed a genome-wide synteny analysis using NGenomeSyn (45), which corroborated the gene order and FISH data by assessing the conserved order of syntenic blocks between species (*SI Appendix*, Fig. S4*G*).

### Northern and Southern White Rhinoceros Genomic Comparison.

**Fig. 3.**
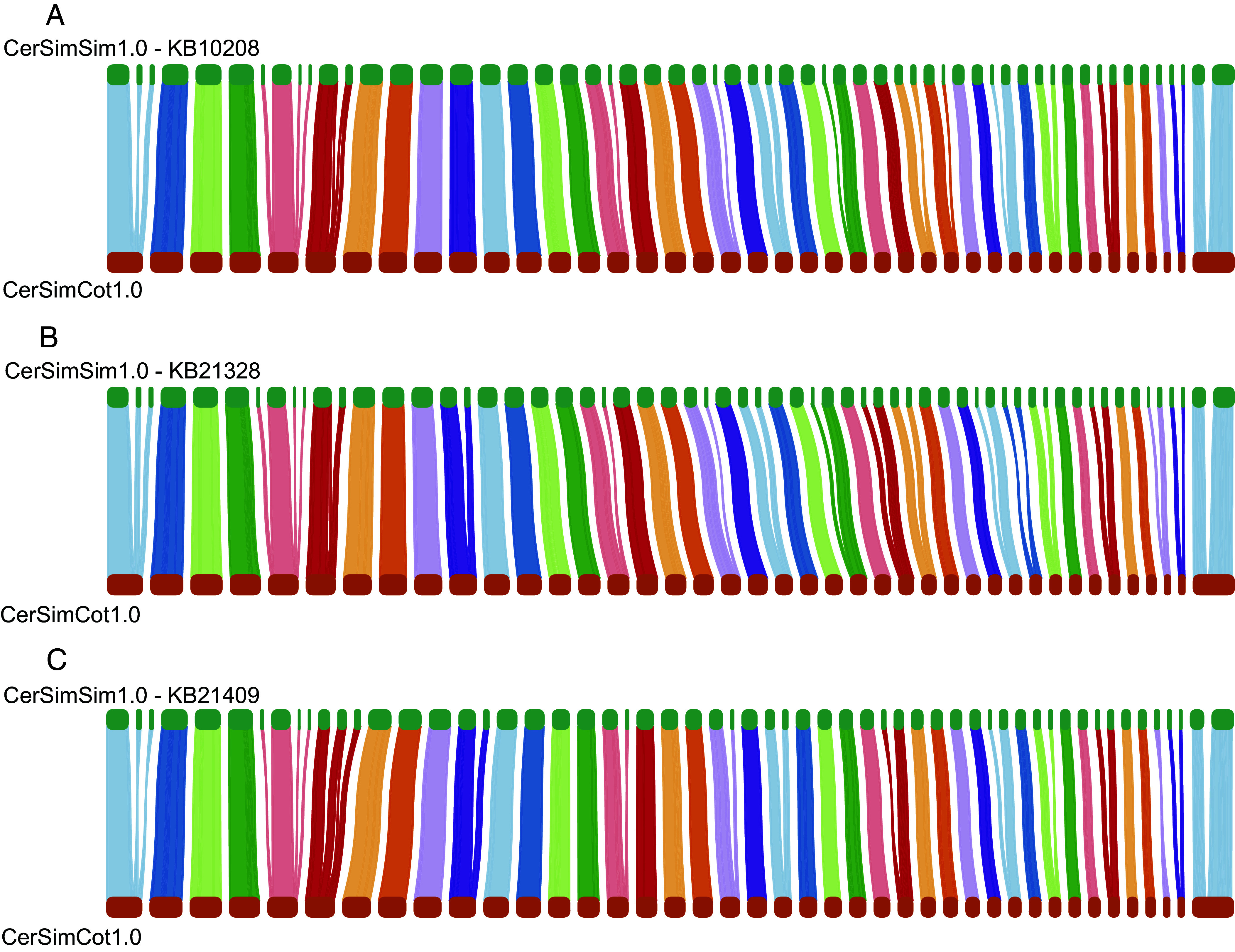
Whole-genome comparison between the NWR (CerSimCot1.0) and three SWR genomes: (*A*) KB10208 “Chuck”, (*B*) KB21328 “Amani”, and (*C*) KB21409 “Wallis”. Synteny is shown with the NWR genome. Regions of conserved synteny between the NWR and SWR genomes are indicated by colored blocks, with lines connecting homologous regions. The original SWR genome assembly (CerSimSim1.0) was rescaffolded using optical maps from the SWR individuals.

To explore the genomic similarities and differences between NWR and SWR, we compared the NWR assembly, CerSimCot1.0, with the previously published SWR genome assembly (CerSimSim1.0, GenBank assembly accession: GCA_000283155.1, *SI Appendix*). Initial comparisons revealed several apparent translocations between NWR and SWR (*SI Appendix*, Fig. S6*A*). However, given that CerSimSim1.0 is of significantly lower quality than CerSimCot1.0 (*SI Appendix*, Table S6), we reasoned that these apparent translocations might be artifacts of assembly errors rather than true genomic differences. To investigate this, we employed OGM (OGM; Bionano) data from three SWR individuals (one male and two females) to rescaffold the published SWR assembly using each individual’s specific optical genome map (*SI Appendix*). While this rescaffolding approach did not improve the base-level accuracy or resolve existing gaps within the original CerSimSim1.0 contigs, it significantly improved the contiguity of the assembly, resulting in 64, 66, and 69 near-chromosome-scale scaffolds for the three individuals, respectively, compared to the initial 3087 scaffolds in CerSimSim1.0 (*SI Appendix*, Table S6). Comparisons of these three independently rescaffolded SWR genomes with the NWR CerSimCot1.0 genome assembly (*SI Appendix*) revealed high chromosome-level synteny (Fig. 3 and *SI Appendix*, Fig. S6) with no consistent large-scale structural variations observed across all comparisons. The high degree of synteny observed between the rescaffolded SWR genomes and the NWR assembly strongly suggests that the initial apparent translocations were likely assembly errors in CerSimSim1.0 rather than true genomic differences.

### Genomic Integrity of the NWR iPSC Lines.

Along with broad differentiation potential, a common characteristic of all iPSCs is their unlimited capacity to proliferate. In culture, iPSCs acquire mutations and structural variants that give a selective advantage to the affected cells, changing the genomic characteristics of the population over time, termed “evolution in the incubator” (18). The genomic integrity of human iPSC lines has been intensively studied, using karyotyping, SNP genotyping, genome sequencing, and OGM. Many common mutations and recurring CNVs have been reported, many of which affect differentiation of the cells or increase their propensity to form tumors. The genomic integrity of iPSCs is critical for their use in downstream applications, including human iPSC-derived cell therapies and differentiation into viable cell types. Genomic integrity is critical for rare species’ assisted reproduction to avoid passing on detrimental or lethal abnormalities to offspring.

**Fig. 4.**
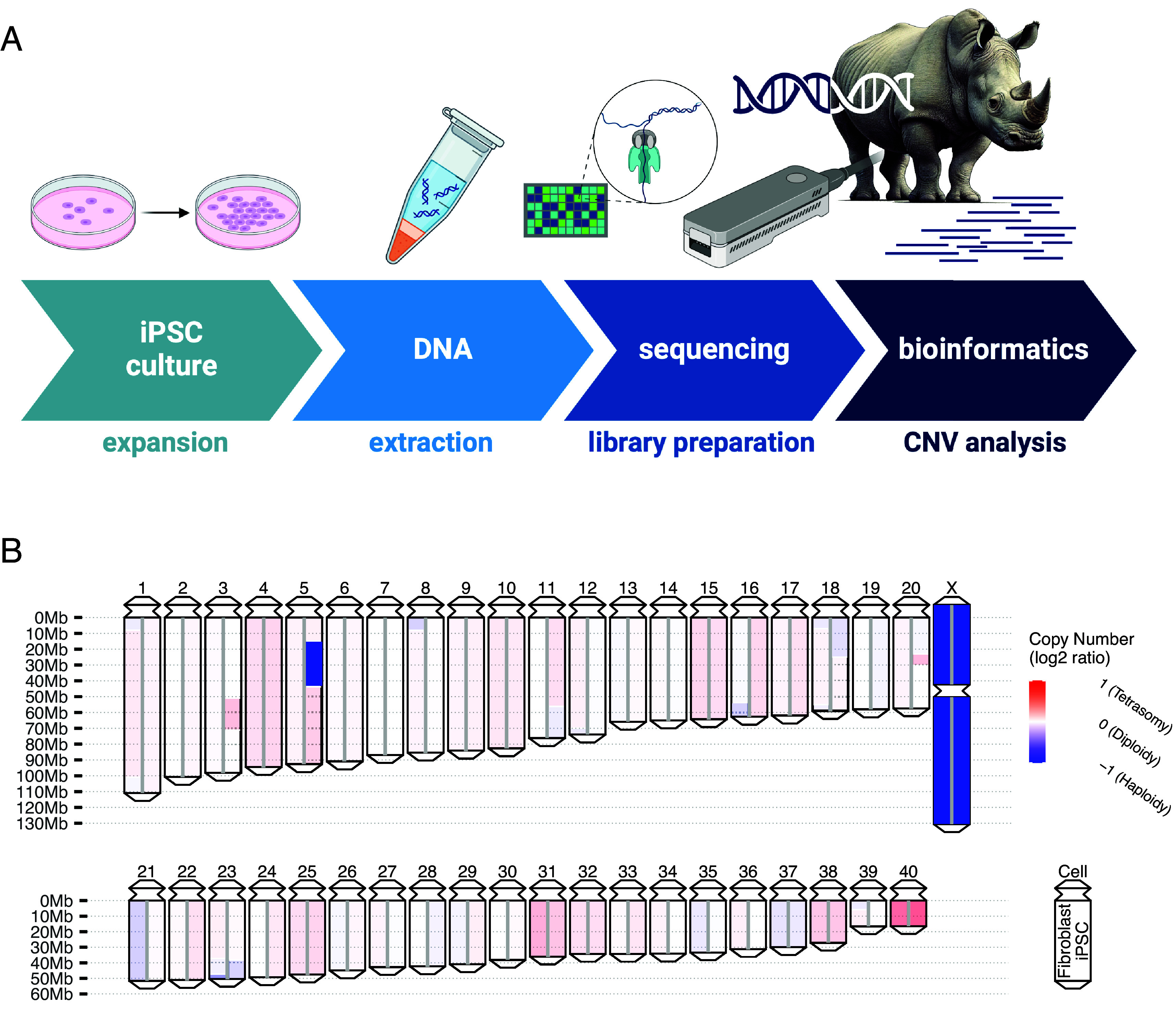
Genomic integrity in cell cultures of NWR. (*A*) A workflow for using nanopore sequencing to assess the genomic integrity of iPSCs lines from endangered species (Created in https://BioRender.com). (*B*) The ideogram of each chromosome shows color-coded values on a log2 scale, from fibroblast cultures on the *Left* half and from passage 40 iPSCs from the same NWR individual (“Angalifu”, lab number KB9947) on the *Right*. Hemizygosity is apparent in a region of chromosome 5 and for the X chromosome in this male animal.

While the existence of reference genomes enables high-resolution detection of chromosomal abnormalities in human cells, most rare species do not have a genomic reference for development of methods such as SNP arrays that are widely used for detecting common variants and CNVs in humans. In order to assess the integrity of the NWR genome, we proposed a workflow for using nanopore sequencing to assess the genomic integrity of iPSCs lines from endangered species. The process includes iPSC culture, DNA extraction, library preparation, nanopore sequencing, and bioinformatics analysis to detect potential CNVs and amplifications and deletions of whole chromosomes (Fig. 4*A* and *SI Appendix*). We adapted an automated bioinformatics pipeline to evaluate the genomic integrity of nanopore reads from NWR fibroblasts and the iPSC line (9947-c501, passage 40) from the same NWR individual using CerSimCot1.0 as a reference genome (*Materials and Methods* and *SI Appendix*). Consistent with the male genome of Angalifu, one X chromosome is detected as haploid compared to the remaining somatic chromosomes of both the fibroblast cultures and iPSCs (Fig. 4*B*).

To assess potential genomic changes that could have been introduced during fibroblast culture, we plotted CNV profiles from a previously published short read dataset obtained at passage 2 (9) with short read data obtained with 10XG at passage 6, HiC short read sequencing at passage 6, and Nanopore long read sequencing at fibroblast passage 12. The comparison indicates that no detectable culture-induced changes had occurred on fibroblast in vitro culture from passages 2 to 12 (*SI Appendix*, Fig. S7 *A–C*).

In the comparison of the iPSC line with the primary fibroblasts from which it was derived, we observed a heterozygous 30 Mb deletion on chromosome 5 (Fig. 4*B* and *SI Appendix*, Fig. S7 *D* and *E*). Because rhinoceros chromosomes are acrocentric and relatively small, identification of copy number variants using karyotyping is far more difficult than it is for primates (Fig. 1*B* and *SI Appendix*, Fig. S4*F*). The alignment of the deleted region of NWR chromosome 5 to the human reference genome suggested that this region corresponds to human chr3p24.3 and chr3q23-24. Recurrent hemizygous deletions in this region have been reported in multiple independent human iPSC lines and embryonic stem cell cultures (18), suggesting that deletion of this region in the NWR, which contains more than two hundred protein-coding genes in our genome annotation, may give cells a selective advantage in culture.

## Discussion

Since the first reference genome assembly of phage in 1976, well-annotated genomes have become fundamental to scientific progress in biology. The human genome sequencing project, initiated in 1990, continues to be refined (46), and the first complete sequence of the human Y chromosome was reported only recently (33). Human and model organism reference genomes have enabled enormous progress in determination of gene function, identification of novel disease-associated mutations and structural variants, gene correction and modification, and development of cell and gene therapies. Human pluripotent stem cell (PSC) research and development of clinical applications for stem cells continue to be critically dependent on a current human reference sequence.

The NWR reference genome will enable the types of molecular genetic research in the white rhinoceros that have become standard for human and mouse stem cell scientists: characterization of gene expression profiles of undifferentiated and differentiated PSCs, DNA methylation profiling, generation of reporter lines to develop directed PSC differentiation methods, gene targeting, and sequencing to detect acquired genomic aberrations. These methods will be essential for the generation of new NWR individuals using assisted reproduction technologies and artificial gametes.

NWR and SWR have been separated for tens of thousands of years (9, 23). While the NWR population has declined to only two nonreproductive females, the SWR has been far more successful, becoming the most prevalent rhinoceros population after only approximately 100 individuals survived at the turn of the 20th century (47). Genetic diversity was not a limiting factor in the SWR’s resurgence; interestingly, the genetic diversity of the SWR population as measured by heterozygosity surpasses that of humans (9). For the NWR, there may be sufficient genetic diversity among the cryopreserved cells for reestablishing a sustainable population (9).

An international effort to genetically rescue the NWR (6) proposed a systematic list of approaches to generate NWR embryos that could be gestated in SWR as surrogate mothers, including somatic cell nuclear transfer (SCNT) using SWR oocytes as recipients of NWR nuclei, fertilization of oocytes recovered from the two females with NWR sperm, or generation of artificial gametes from the collection of iPSC lines from NWR individuals (8, 12–15, 48).

The chromosomal number of SWRs and NWRs is identical (24) and our analysis indicates that there are no large structural differences between their genomes. In 1977 a hybrid offspring (“Nasi” KB5767 SB #476) was born in captivity to a NWR mother (“Nasima” KB8174 SB # 352) and a SWR father (“Arthur” SB # 355) (49). Nasi failed to reproduce during her 30 y lifespan, but the reasons are unknown. The similarity between the genomes supports some of the ideas discussed in the genetic rescue proposal (6); for example, if SWR mitochondria are compatible with NWR nuclei, SCNT may gain support as a viable option.

Critical to any successful genetic rescue effort involving iPSCs as an unconventional source of gametes is the rigorous quality control of stem cell lines and their differentiated derivatives using efficient genomic methods. As demonstrated in this work, a highly contiguous reference genome allows the use of shotgun sequencing data to assess the genomic integrity of PSC cultures (50). Lacking the cytogenetic tools that give human karyotyping and SNP genotyping sufficient resolution for detection of CNVs (18, 51), we instead used nanopore sequencing and structural analysis to look for aberrations in the NWR genome and detected a 30 Mb hemizygous deletion in an NWR iPSC line. Since this variant was not detected in the parental fibroblasts, we assume that it arose during expansion of the iPSCs, as has been shown for human iPSCs (52). The deletion results in the heterozygous loss of more than 200 genes in our genome annotation, including seven genes with human homologs involved in various stages of meiosis and germ cell function and two tumor suppressor genes (*SI Appendix*, Table S8). Although this particular deletion is not identical to those found in human cancers, similarly large heterozygous deletions have been frequently observed in malignancies, such as the loss of a 25 Mb section of chromosome 19q in brain tumors (53). Given the potential impact of such extensive genomic alterations on cell function, this type of deletion may represent a significant genomic scar, making the affected iPSC line unsuitable for generating gametes for genomic rescue.

We recognize that a perceived limitation of CerSimCot1.0 may be that it was assembled from cultured cells instead of primary tissues. But in many cases, cultured cells are the only source of living material from a particular deceased animal. This is in large part due to historically inadequate funding, facilities, and trained personnel for cryobanking rare species. Remarkably, in 1975, the founders of San Diego’s Frozen Zoo had the foresight to generate living fibroblast cultures from tissues collected in the wild and from zoos, which enabled our successful generation of iPSCs from 7 deceased NWRs as well as the two remaining alive. We would encourage future banking efforts to, if possible, preserve both cultured cells and primary tissues so that sufficient DNA can be obtained from animal tissues to make use of newly developed genomic technologies that require large amounts of material.

We conclude that endangered species iPSCs will need to be subjected to genomic analysis to confirm their genomic integrity. This will enable conservation scientists to avoid iPSC clones with significant genomic alterations similar to those we observed in the iPSC line we studied here, instead choosing clones without deleterious mutations for generation of gametes or other cell types.

Our findings suggest that iPSCs from many mammalian species will have an inherent propensity to acquire cell culture-induced genomic abnormalities. For other rare animals, we propose that it will be most efficient to first assemble and validate a high-quality, chromosome-level genome and then use inexpensive short and long-read sequencing datasets from individual iPSC lines to assess their genomic integrity. We propose an integrated general workflow for doing this, in which a rapid sequencing method could be used in conjunction with the reference genome to identify genomic abnormalities (*SI Appendix*).

We evaluated this approach, using our reference genome to analyze another iPSC line (9939-c5101) from another NWR individual (“Saut,” * September 1972 (est) - † August 14, 2006, laboratory number KB 9939). Using rapid ONT sequencing methods we detected amplifications in chromosome 2 and chromosome 17 (*SI Appendix*, Fig. S8). We recommend this approach for iPSC lines in any laboratory working on deriving gametes or other cell types from endangered species.

Beyond the work reported here, once a contiguous reference genome has been established, future work in stem cell quality control for nonmodel mammals could focus on the development of efficient bioinformatic assays to evaluate pluripotency (54) and differentiation potential (55) of such cell lines toward further optimized protocols for the derivation of viable gametes from iPSCs (15).

Our approach will also aid in the assembly of genomes from other endangered rhinoceros, and understanding genetic relatedness may support the implementation of population-scale strategies such as managed breeding.

## Materials and Methods

Detailed Methods for many of the procedures are provided in **SI Appendix**, as indicated below.

### Sample Collection.

A wild-caught male NWR was chosen as the reference individual: Angalifu, lab ID KB9947 and studbook # 348. This individual was selected because it was previously identified (9) as a male with high runs of homozygosity relative to the NWR population to facilitate the assembly process. Fibroblast cell lines were obtained from the Frozen Zoo® part of the Wildlife Biodiversity Bank at the San Diego Zoo Wildlife Alliance and reprogrammed into iPSCs (iPSCs, cell line NWR 9947-c501) as previously described (8). These cells were then expanded, harvested, and cryopreserved as needed for each sequencing application.

### Genomics and Bioinformatics.

The methods for DNA and RNA extraction, genomic library preparation, sequencing, genome assembly, genome size estimation, chromosome assignment, genome quality evaluation, and genome annotation are provided in *SI Appendix*.

### FISH Mapping.

The genes highlighted in Fig. 2 have supporting mapping from FISH mapping experiments on equine chromosomes and with at least one rhinoceros species (greater one-horned, southern white, northern white, southern black, eastern black, and Sumatran rhinoceros).

Metaphase spreads for each species were prepared as previously described from rhinoceros fibroblasts obtained from the Frozen Zoo^®^ (24). Domestic horse BACs that were previously mapped were obtained either from the CHORI BACPAC library (56) or INRA (57), expanded, DNA extracted, and labeled with Spectrum Green, Red, or Orange (Vysis) as previously described (58). In situ hybridization was performed for 72 h with 50 to100 ng of each probe, 4 mg of horse competitor DNA, and 6 mg of species-specific rhinoceros DNA. 71 horse probes were successfully cross-hybridized in the rhinoceros species (*SI Appendix*, Table S7).

Gene IDs for each BAC clone were obtained from the published literature and identified either through overgo probes or BAC end sequencing (BES). We have used updated naming conventions for mapping purposes for those genes that have changed their designation since the publication of the respective BACs (bold genes in *SI Appendix*, Table S4). In some cases, the published gene was misidentified due to the short sequence alignment of the overgo probes. For these cases, we used BLAST with the original sequence when available against EquCab3.0 to obtain the correct gene for each BAC. BACs for RPS6 (INRA-0326E11) and GNMT (INRA-228G6) were eliminated from the data because the correct gene name could not be identified even though these genes can be mapped well to EquCab3.0. The coordinates of each BAC in relationship to the horse genome were obtained from the most recent assembly of the equine genome (EquCab3.0) and aligned to our NWR assembly. The horse transcriptome was mapped to the NWR genome assembly, and a detailed comparison between the NWR and SWR genomes was performed (see *SI Appendix* for a complete description of these analyses).

### Genomic Integrity of iPSC Lines.

The generated CerSimCot1.0 reference genome was evenly split into bins of 1 Mbps since we were interested in chromosomal-level abnormalities in the iPSC lines. We aligned the nanopore reads to the reference genome using Minimap2 (59) with parameters -ax map-ont and kept primary alignments using Samtools (60) with parameter -F 2308. We counted the number of reads aligned to each bin. Bins with read counts higher (lower) than expected values indicate amplifications (deletions). The somatic copy number of each bin was inferred by the log2 value of the normalized read counts in each bin. Neighboring bins with similar copy number values were merged into segments using the circular binary segmentation algorithm (61). This method was applied to the nanopore reads of fibroblast lines and iPSC lines in which the genomic stability results of fibroblasts were used as a control to validate the genomic stability of iPSCs.

### Workflows for Culture and Genomic Analysis.

Detailed workflows are provided in *SI Appendix*.

## Supplementary Material

Appendix 01 (PDF)

## Data Availability

Final assembly and sequencing data are available in NCBI Bioproject PRJNA734732 (62). The pipeline used to evaluate the genome integrity of NWR iPSC lines is available on GitHub: https://github.com/GJYWang/NWR_iPSC.git (63).
