# Supplementary material for: Genomic map of the functionally extinct northern white rhinoceros (Ceratotherium simum cottoni)
Source: Proc Natl Acad Sci U S A. 2025 May 13;122(20):e2401207122. doi: 10.1073/pnas.2401207122 (PMC12107126; doi:10.1073/pnas.2401207122)
Supplement: Supplementary file 1 — Appendix 01 (PDF) [file pnas.2401207122.sapp.pdf]

Supporting Information for

Genomic map of the functionally extinct northern white rhinoceros (*Ceratotherium simum cottoni*)

Gaojianyong Wang[1*], Marisa L. Korody[2* #], Björn Brändl[3], Camilo Jose Hernandez-Toro[1], Christian Rohrandt[4], Karl Hong[5], Andy Wing Chun Pang[5], Joyce Lee[5], Giovanna Migliorelli[6], Mario Stanke[6], Sarah Ford[2], Iris Pollmann[4], Marlys L.Houck[2], Harris A. Lewin[8], Teri L. Lear[9 $], Oliver A. Ryder[2], Alexander Meissner[1], Jeanne F. Loring[10 #], Franz-Josef Müller[1, 3 #]

1. Department of Genome Regulation, Max Planck Institute for Molecular Genetics, Berlin, Germany
2. San Diego Zoo Wildlife Alliance, Escondido CA USA
3. Department of Psychiatry and Psychotherapy, Christian-Albrechts University, Kiel, Germany
4. Institute for Communications Technologies and Embedded Systems, Kiel University of Applied Sciences, Kiel, Germany
5. Bionano Genomics Inc, San Diego, CA USA
6. Institute of Mathematics and Computer Science, and Center for Functional Genomics of Microbes, University of Greifswald, Germany
7. The Genome Center, University of California Davis, Davis, CA USA
8. Department of Ecology and Evolution, and John Muir Institute for the Environment, University of California Davis, Davis, CA USA
9. M. H. Gluck Equine Research Center, Department of Veterinary Science, University of Kentucky, Lexington, KY USA
10. Scripps Research, La Jolla, CA USA

*These authors contributed equally to the work.

$ posthumous publication of work conducted from 2002 – 2010

# Co-corresponding authors, JFL: jloring@scripps.edu; FJM: fjmuellr@molgen.mpg.de; MLK: MKorody@sdzwa.org.

This PDF file includes:

Supporting text
Figures S1 to S8
Tables S1 to S8
SI References

Supporting Information Text

SI Materials and Methods

DNA Extraction, Genomic Library preparation, and sequencing

10X linked reads: 1.5 x 10^6 fibroblasts (passage 6) were harvested using 0.05% Trypsin EDTA (Gibco), rinsed once with DPBS, and flash-frozen in liquid nitrogen. Frozen cells were then provided to the DNA Technologies and Expression Analysis Core at the UC Davis Genome Center (supported by NIH Shared Instrumentation Grant 1S10OD010786-01) for high molecular weight DNA extraction library preparation and sequencing.

HiC sequencing: 1.5 x 10^6 fibroblasts (passage 6) were harvested using 0.05% Trypsin EDTA (Gibco), rinsed once with DPBS, and flash-frozen in liquid nitrogen. Frozen cells were then provided to Dovetail™ Genomics (Scotts Valley, CA) for extraction, library preparation, and sequencing.

Bionano Genomics: 1.5 x 10^6 fibroblasts (passage 8) were harvested using 0.05% Trypsin EDTA (Gibco), pelleted at 0.3g for 5 minutes, and resuspended in DMEM supplemented with 10% FBS and 10% DMSO. Cells were then slow-cooled in a Corning® CoolCell™ for at least 4 hours to -80 °C before continued short-term storage at -80 °C until processing.

Ultra-high molecular weight DNA was isolated by using an agarose plug method (Bionano prep cell culture DNA isolation protocol #30026 revision F) or a silica disk protocol (Bionano Prep SP Frozen Cell Pellet DNA Isolation Protocol v2 #30398 revision A) to stabilize the DNA during isolation and purification. In short, DNA from 1.5 million cells was treated with RNase A and proteinase K in the presence of detergents and either embedded into an agarose plug or bound to a 4mm silica disk. The bound or embedded DNA was washed, eluted, and allowed to homogenize at room temperature overnight before fluorescent labeling. 750 ng DNA was labeled at the recognition site CTTAAG the following day using the enzyme DLE-1. The DNA was counter-stained and imaged on a Bionano Genomics Saphyr Gen II system until at least 400X raw coverage was collected for the genome assembly. At least 100X raw coverage was collected for structural variant detection.

Oxford Nanopore Technologies (ONTs): 30 – 50 x 10^6 fibroblasts (passage 12) or iPSCs (passage 40) were harvested with 0.05% Trypsin - EDTA (Gibco) or Accutase (Corning) respectively, pelleted at 0.3g for 5 minutes, rinsed once with DPBS and resuspended in 100 μL of DPBS for phenol:chloroform extraction [1]. Briefly, cells were incubated for one hour at 37 °C in TBL buffer containing 10 mM Tris-CL, 25 mM EDTA, 0.5% SDS (W/V), and 20 μg/mL of RNase A. Cell suspension was then incubated for 3 hours at 50 °C with 100 μg/mL of Proteinase K, rotating end over end 10 times after each hour. The lysate was poured into a tube containing phase-lock gel, and an equal volume of buffer-saturated phenol was added. Samples were rotated for 10 minutes at 40 rpm and centrifuged for 10 minutes at 4500 rpm. The aqueous upper layer was poured into a new phase-lock tube, adding

an equal volume of buffer-saturated phenol:chloroform. Samples were again rotated and centrifuged for 10 minutes. The aqueous upper layer was poured into a fresh tube, and a 1/10 volume of 7.5M ammonium acetate and 2.5 volumes of cold absolute EtOH were added. DNA was then spooled and rinsed in freshly prepared 70% EtOH. Spooled DNA was washed twice with 80% EtOH and resuspended in 10 mM Tris-Cl pH 8 overnight. DNA concentration was then quantified in triplicate using a Qubit Fluorometer (Thermo Fisher Scientific).

30 μg of DNA was sheared using a Covaris ® g-Tube to 20 kbp by centrifuging for 60 seconds at 7,200 RPM. Alternatively, some DNA was sheared by passing through a 26g needle approximately 5 times. Sheared DNA was then size-selected using a BluePippin (Sage Science) with either a 15 kbp or 20 kbp lid to remove small fragments.

Size selected DNA was then used for the Genomic DNA by Ligation (SQK-LSK109) library preparation (ONT, UK) according to the published protocol with minor adjustments: Up to 6 μg of DNA was used for each library preparation; DNA Repair and End-prep reaction incubation times were increased to 20 minutes at 20 °C and 10 minutes at 65 °C; End-prep reaction AMPure XP bead cleanup elution step was performed for 25 minutes at 60 °C with flicking every five minutes; Adaptor ligation was performed for 30 minutes at room temperature; AMPure XP beads were then incubated for 30 minutes at 48 °C with flicking and washed with "Long Fragment Buffer;" 80% EtOH was used throughout for all washing steps.

Sequencing was performed on a MinION instrument using R9.4.1 flow cells. Approximately 700ng of g-Tube sheared or 1.6 μg of needle sheared prepared library was loaded for each flow cell, and washes were performed after 20-24 hours using the Flow Cell Wash Kit (EXP-WSH003) and reloaded with a fresh library for a total run time of 72 hours.

RNA Extraction, Library Preparation, and Sequencing

RNA Extraction: Cells were removed from the plate using either 0.05% Trypsin - EDTA (fibroblasts) or Accutase (iPSCs) as previously described, washed once in DPBS, and resuspended in 600 μL of lysis buffer from *mir*Vana™ miRNA Isolation kit (Invitrogen) and stored at -80 °C until extraction. Tissue was collected and stored at -80 °C until use, when it was finely minced on dry ice using a scalpel in the presence of lysis buffer. RNA was then extracted from the cells or tissue following the manufacturer's instructions. RNA was quantified using the Qubit fluorometer, and 100ng of total RNA was used for the library preparation.

Library Preparation: Total RNA was Poly(A) selected using the NEBNext® Poly(A) mRNA Magnetic Isolation Module as part of the library generation protocol for the NEBNext® Ultra™ II Directional RNA Library Prep Kit for Illumina® (New England Biolabs, Ipswich, MA) following the manufacturer's instructions. RNA fragmentation was chemically performed at 94 °C for 15 minutes for a target insert size of 200 bp, and the PCR enrichment step

was performed for 12 cycles. Library concentrations were determined using the Qubit, and insert sizes were obtained using the DNA High Sensitivity Bioanalyzer (Agilent Technologies) reaction and using these values diluted for sequencing. 76bp paired-end sequencing was performed on an Illumina NextSeq 500 by the Sanford Burnham Prebys Genomics Core (La Jolla, CA).

NWR genome assembly

Contig Generation: Guppy4 (ONT, UK) was used to base-call the ONT raw reads, and Shasta 0.5.1 [2] was used to generate the contigs from the ONT reads using parameters `--memoryMode filesystem --memoryBacking 2M –threads 128` for optimal performance. The contigs generated by Shasta are referred to as primary contigs (ct).

10X scaffolding: Scaff10X v4.2 [3] was used to compute an adjacency matrix after the 10X Genomic linked reads had been aligned to ct. One round of scaffolding was performed with the parameters `-nodes 63 -read-s1 12 -read-s2 8 -link-s1 10 -link-s2 10`. The generated scaffolds were referred to as scaffold1 (sf1).

Bionano scaffolding: Bionano Solve 3.3 (Bionano Genomics, Inc.) was utilized to scaffold sf1 with the haplotype-unaware optical genome mapping (OGM) molecules obtained from "Angalifu". The scaffolding process was performed with the parameters: pre-assembly ON, extend and split OFF, and cut complex multi-path regions ON. The scaffolds generated with the Bionano optical maps were referred to as “scaffold2” (sf2).

HiC scaffolding: The mapping pipeline from Arima Genomics [4] was used to align Hi-C reads to the scaffold sf2. In the mapping pipeline, both ends of a read pair were individually aligned to sf2 using BWA-MEM [5] with default parameters. Only the 5’-side of the chimeric reads mate-pair was retained, and the 3’-side was removed from the two bam files generated by BWA-MEM in the previous step. Next, a sorted and filtered bam file of paired single-end HiC reads was generated from combining the two bam files and filtering out the reads with mapping quality (MAPQ) less than 10. Picard Tools [6] were used to remove PCR duplicates in the pair-end bam file, the last step of the Arima Genomics mapping pipeline. Salsa2 [7] was used to estimate the orientation and location of contigs based on the normalized frequency of HiC interactions between them. Five rounds of scaffolding were performed with the capability to identify misassemblies in sf2 using parameters `-m yes -i 5 -p yes`. The restriction enzyme for Arima HiC data is set by parameter `-e GATC`. After 5 rounds of scaffolding, the generated scaffolds were referred to as “scaffold3” (sf3).

Polishing: One round of Racon polishing [8] and one round of Pilon polishing [9] were performed using HiC and 10X linked reads. Using bam files generated from the HiC reads and 10X linked reads aligned to sf3, Racon was performed with default parameters. Pilon was performed with parameter `java -Xmx512G -jar pilon-1.23.jar` using bam files generated from the HiC reads and 10X linked reads aligned to the polished scaffolds from Racon using BWR-MEM [5]. The ONT reads were aligned to the polished scaffold from pilon, and all

scaffolds with mean coverage lower than 40 or higher than 200 were removed. After polishing and scaffold removal, the resulting scaffolds were called “polished scaffolds” (ps).

Juicebox curation: We then applied the Juicer [10] and 3D-DNA [11] pipeline to ps for manual curation visualizing ps using Juicebox [12], correcting minor errors in ps, and combined ps into 41 scaffolds, referred to as the first draft of the rhinoceros assembly (fd).

Allosome assembly: We aligned all the nanopore reads to fd using minimap2 [13] and obtained the coverage profile of each scaffold. The coverage histogram of one scaffold, corresponding to a hybrid allosome, contains two peaks centered at 38 and 75. In contrast, the coverage histograms of the remaining 40 scaffolds, corresponding to the autosomes, contained only one peak centered at 75. Because only one copy of chromosome X and one copy of chromosome Y exist, those regions in the hybrid allosome with the same coverage as the autosomes are regions present in both X and Y chromosomes.

We identified all the bases in the hybrid allosome with coverage values between 56 and 81. Continuous DNA sequences bounded by any of the identified bases with a distance of less than 50 bps were selected out. All the selected DNA sequences longer than 100 bps were called segments in CHR Y (sgcy). Sgcy is aligned to each nanopore read. In order to find the reads coming from chromosome Y, we selected out all the reads with more than 5% bases overlapping with sgcy. Canu was used for the assembly of the selected reads, referred to as CHR Y contigs (cty1).

We reasoned that the Y-chromosome-reads selection process described above may still contain reads from chromosome X and other autosomes. These reads from chromosome X or other autosomes would probably remain either unassembled or assembled with low coverage in Canu [14]. Even if these reads could be assembled into a few contigs with normal coverage, these few contigs would not bear any resemblance to sgcy. Following this logic, we 1) aligned the selected reads sgcy to cty1 and 2) aligned sgcy to cty1. Also, we removed a contig in cty1 if this contig met the following two criteria: 1) average coverage of the contig is lower than low, and 2) less than 5% of the bases in the contig overlap with sgcy. We generated contigs of a total size of 46 Mbps, referred to as cty2.

Cty2 was scaffolded by 10X linked reads [3] and HiC sequencing [4], similar to the scaffolding of the whole nuclear genome. The generated scaffold is called sfy1. Next, sfy1 was scaffolded using Bionano Solve 3.6 (Bionano Genomics, Inc.) by aligning sfy1 to the Bionano OGM molecules. The algorithm iteratively extended the NGS contigs using Bionano's long molecules and merged any overlapping contigs resulting from the extended length. The generated scaffold from Bionano is called sfy2. Finally, sfy2 was polished once using Racon [8] and Pilon [9] to generate CHR Y scaffolds (ys).

We aligned ys back to the hybrid sex chromosome. The regions where the hybrid sex chromosome overlaps with ys should be either DNA sequences in both chromosomes X and Y or DNA sequences in chromosome Y but are misassembled into chromosome X. The DNA sequences that exist in chromosome Y but are misassembled into chromosome X should have a coverage half of the coverage in autosomes. Therefore, we trimmed the hybrid sex chromosome where it overlaps with ys and with low coverage (less than 38) to generate an updated version of chromosome X. The updated X and Y chromosomes and the autosomes in the first draft were merged to create the second draft of the rhinoceros assembly (sd).

Mitogenome assembly: All the ONT reads were aligned to the mitogenome of domestic horse (*Equus caballus*, EquCab3.0, GenBank assembly accession: GCA_002863925.1 [15]) using minimap2 [13]. Since a mitogenome is usually less than 19kb, those reads whose length was less than 19kb and whose MAPQ was larger than 30 were selected. Only reads with aligned length larger than 1 kbp were included. These selected reads were assembled into a contig of 33kb using Canu [14]. The front of this contig was identical to its rear, suggesting it should represent a circular genome, which was then manually trimmed to remove the overlap. After manual trimming, a contig of 16,715 bp was obtained, representing the complete mitogenome of "Angalifu". The assembly process was also performed with the less closely related mitogenome of *Balaenoptera musculus* (blue whale) [16] yielding an identical mitogenome of 16,715 bp. The mitochondrial genome was then corrected by ONT reads through manual curation. The mitogenome (mt) was merged to sd to generate the third draft of the rhinoceros assembly (td).

Nuclear mitochondrial DNA (NUMT) segment identification: We used a similar approach as described in [17]. We used k-mer search instead of BLAST. We collected all the 21-mers from the mitochondrial genome (MT) and mapped these 21-mers to the nuclear genome assembly with a maximum 2 mismatches or indels. The mapped regions on the nuclear genome assembly represent the NUMTs. We then collapsed the identified NUMTs into larger blocks if they were separated by less than 20 bps.

NUMTs and MT Methylation analysis: CpG methylation status were basedcalled for all reads by Guppy (ONT, UK). Since MT is circular, we concatenated two identical MT sequences into one continuous sequence to allow the alignment tool to map entire MT reads without secondary alignment (primary alignments were kept using samtools with parameters `-F 2308`) for analysis. Methylation rates were obtained using Modkit (ONT, UK). Methylation levels were then plotted for the three largest NUMTs and MT.

Gaps and manual curation: We aligned all the ONT reads to td using minimap2 [13] and curated the alignment results in Integrative Genomics Viewer [18]. The ONT reads covering both ends of a gap were used to close the gap. The final assembly after the curation of td was called the final draft of the rhinoceros assembly. Additional manual curation of the Y was performed by aligning female RNAseq data to the Y-scaffolds and identifying

scaffolds that could not be located on the X or Y. These scaffolds were manually examined, blasted, and subsequently renamed as unlocalized scaffolds.

Genome size estimation

We used Meryl [19] to count 31-mers from the ONT reads and generate histograms from these reads so as to estimate genome size, repeat content, and heterozygosity using GenomeScope [20].

Metrics of genome assembly quality:

Continuity: 1) The contig/scaffold NG50, defined as the size of a contig/scaffold where the sum of all the contigs/scaffolds with sizes longer than this scaffold is larger than half of the estimated genome size, is the current standard in the measure of genome assembly continuity. The northern white rhinoceros assembly scaffolds have reached a Chr.NG50, which represents the highest standard in genome assembly. 2) Gaps, given by the number of gaps existing in the final assembly, quantify the number of unresolved regions in the genome.

Base accuracy: 1) Base pair QV was estimated by a reference-free method integrated into the Merqury pipeline [19]. Since k-mers uniquely identified in the assembly and not shown in the raw data are caused by errors, the assembly k-mers only existing in the genome assembly can be used for base-pair QV estimation. 2) K-mer completeness, obtained using the Merqury pipeline [19], is given by the k-mer found in an assembly divided by the k-mers found in the raw read data.

Structure accuracy: 1) False duplications: If a genome is wholly sequenced with coverage of *c*, then a unique k-mer from a homozygous (diploid) or heterozygous (haploid) region can be found *c* or *c/2* times. False duplications due to the genome assembly process can be estimated by counting extra k-mers. We used Merqury [19] to count the number of distinct k-mers in the genome with additional copies and estimated the ratio of false duplications. 2) Reliable blocks were given by the concordance of at least two types of sequencing data out of four (ONT, 10X linked reads, Bionano, and HiC) supporting the assembled structure at each base [21].

Haplophasing: We aligned the pair-end HiC reads to the NWR genome using BWA-MEM [5] with default parameters. Freebayes [22] was used to call variants from the HiC-short-read bam file with coverage below 30 (i.e., `-g 30`). We aligned the ONT reads to the final draft of the northern white rhinoceros genome using minimap2 [13]. Whatshap [23] was used to phase the identified variants and to generate haplophased blocks using the generated ONT-long-read bam file with default parameters.

Functional completeness: BUSCO (Benchmarking Universal Single-Copy Orthologs) [24] V5.1.2 was used to evaluate the functional completeness of the generated assemblies. Assemblies were compared with lineage datasets `eukaryota_odb10` containing 255 genes, vertebrata_odb10 containing 3554 genes, and `laurasiatheria_odb10` containing 12234 genes.

Telomere and centromere assessment: The telomere sequence (TTAGGG) is conserved across various species [25]. To identify the centromere sequence of the NWR, we downloaded the Illumina short-read WGS sequencing data (SRR11428438) of a NWR from NCBI. We randomly sampled 100k reads from this dataset and assembled the sampled reads into contigs using PRICE (Paired-Read Iterative Contig Extension) assembler [26]. The repeat sequences in the generated contigs were identified using TRF (Tandem Repeat Finder) [27]. This resulted in the identification of a 228bp repeated sequence, which was confirmed in our generated genome assembly:

TTTGGCTCAGTTCTGGGAATCTTTGAACGTCCTATACAAACTTGTTCCTAGATAGTAACTTGCAGCTT
GGAATGAGAAAGTGCTTGCTTACAGCCAAGTGCTGTATGTTCTGAATGCCCGCATTGGGAATCTGTG
TCAAAACTTTGTTAGGAATTGTGTGATCCTTACTAGCTGGAAGTAAGGGAGATGGGGCATGAAGTTA
TTTCTAGGAGTTGGAGACATACCCAC

Mapping of horse transcriptome to NWR genome

The complete transcriptome in the current horse reference genome was aligned to the NWR genome using BWA-MEM [5], and the coordinates of these alignment results were extracted. We used a scoring matrix (each row represents a horse chromosome, and each column represents an NWR chromosome) to trace the gene mapping relationships between horse and NWR: 1) a horse gene from any horse chromosome had one point, and 2) this point was distributed equally to the NWR chromosome to which this gene was aligned. We normalized the scoring matrix by row and obtained the percentage of genes on each horse chromosome aligned to the NWR chromosomes (**Fig. 2** and **Fig. S3a**).

Chromosome assignment

The X and Y chromosomes have been defined in previous sections. Consecutive integer numbers were assigned to the remaining scaffolds based on their sizes, i.e., the longest scaffold corresponds to chromosome 1, while the shortest scaffold corresponds to chromosome 40.

Softmasking genome

The final draft genome was soft masked using RepeatMasker [28] with the command `RepeatMasker -pa 15 -qq -species mammal -gff -xsmall -nolow`

Genome and functional annotation

The RNA libraries were aligned to the NWR assembly using the STAR aligner [29]. We ran BRAKER3 [30] using the generated alignment files and protein datasets with pre-trained AUGUSTUS parameters of human, i.e. `--species=human --softmasking –skipAllTraining`; all the predicted genes or transcripts were blasted and functionally annotated against the Swiss-Prot dataset from the UniProt Knowledgebase [31].

Synteny analysis

Reference genomes are mapped using Minimap2 [13] with parameters -x asm5. The generated results were visualized using NGenomeSyn [32] with default parameters.

Comparison between NWR and SWR genomes

The generated CerSimCot1.0 reference genome and the SWR reference genome (CerSimSim1.0, GenBank assembly accession: GCA_000283155.1) were compared using Minimap2 [13] with parameters -x asm5. The generated results were visualized using dotPlotly [33] with parameters `-m 2000 -q 500000 -l -p 12`. Several translocations and inversions were identified between CerSimCot1.0 and CerSimSim1.0. To determine whether these structural variants were due to assembly errors or represented actual biological differences, we used Bionano optical mapping to scaffold DNA from three SWRs with CerSimSim1.0.

Scaffolding of SWR genome

Hybrid scaffolding of the SWR genome assembly (CerSimSim1.0, GenBank assembly accession: GCA_000283155.1) was performed using the "resolve all conflicts" parameter, which resolves conflicting alignments between CerSimSim1.0 scaffolds and optical genome maps by assessing molecule support at the conflicting region. If the optical genome map was supported by molecules that span the conflicting region, then the CerSimSim1.0 scaffold was cut and re-scaffolded. If the optical genome map did not have molecule support that spanned the conflicting region, the map was cut and re-scaffolded with the CerSimSim1.0 scaffold (Bionano Solve Theory of Operation: Hybrid Scaffold #30073 revision F).

Comparison between NWR and the scaffolded SWR genomes

The generated CerSimCot1.0 reference genome and the scaffolded SWR reference genomes were compared using Minimap2 [13] with parameters -x asm5 and visualized using dotPlotly [33] with parameters `-m 2000 -q 500000 -l -p 12`.

SI protocol

Protocol for routine genomic QC of iPSCs from endangered species

Introduction

This protocol outlines a workflow (**Fig. 4a**) for using nanopore sequencing to assess the genomic integrity of induced pluripotent stem cell (iPSC) lines from endangered species. The process includes iPSC culture, DNA extraction, library preparation, nanopore sequencing, and bioinformatics analysis to detect potential copy number variations (CNVs) or amplifications and deletions of whole chromosomes. The genomic integrity of iPSCs is relevant for their use in downstream applications, including differentiation into viable cell types such as gametes. The graphical abstract summarizes the workflow, starting from iPSC culture through bioinformatics analysis.

For many endangered species, karyotyping presents significant challenges due to the limited availability of biological material and the lack of comprehensive cytogenetic studies that are typically available for species like humans, laboratory mice, and horses. The standardization of banded karyotypes across populations within a species is labor-intensive and requires extensive sampling, which is often unrealistic for endangered species with small or fragmented populations. However, advances in genomics enable the generation of high-quality, chromosome-level genomes as described in the manuscript. This allows for a more accessible and standardized approach to genome evaluation through sequencing-based methods. By using protocols like the one outlined here it is possible to rapidly, efficiently, and relatively cost-effectively assess the genomic integrity of iPSCs.

This workflow (**Fig. 4a**) serves as a proof of concept for applying nanopore sequencing to the quality control of cultures from non-model species. While the exemplified coverage-based CNV detection is effective at identifying larger-scale genomic abnormalities, increased sequencing depth, particularly with long-read technologies, could be used to uncover more complex structural rearrangements, such as inversions and translocations. Implementing these even more advanced bioinformatic workflows will also require a better understanding of population-scale structural variants, which is currently lacking even for species as well-studied as humans. While this protocol offers a cost-effective and expedient solution for genomic integrity screening, further progress will depend on accumulating broader population-level data for endangered species.

The generation of a high-quality, chromosome-level genome enables the workflow outlined here. As described in the manuscript, such a reference genome provides the foundation for accurately aligning sequencing reads and detecting genomic abnormalities, allowing for precise binning and segmentation relevant to the resolution of CNV detection.

1. iPSC Culture and Expansion:

iPSCs are cultured and expanded to a sufficient number of cells for analysis. Details on the culture conditions, media compositions, and passaging methods for "primed" pluripotent stem cells suitable for the culture of SWR and NWR, can be found in the Methods section of this manuscript and previous publications by us [34, 35] and others [36, 37]. Culture conditions for "naïve-like" iPSC from NWR have been reported recently [38]. The specific culture conditions used for iPSCs may have an effect on their differentiation into the cell types of interest.

2. DNA Extraction:

In this study, high-purity DNA was extracted from iPSCs using standard protocols. The extracted DNA was quantified and quality-checked, following the detailed methods provided in the Methods section.

Notably, nanopore sequencing, combined with ultra-rapid DNA extraction methods, has recently been used for intraoperative diagnosis of brain tumors [39, 40]. With these optimized methods, DNA can be prepared in less than 30 minutes. In a routine laboratory setting, we have found that these methods are suitable for effective and time-saving DNA extraction for this workflow.

3. Library Preparation:

The library preparation step involves converting extracted genomic DNA into a format suitable for nanopore sequencing. This involves end repair and adapter ligation with the conventional nanopore ligation sequencing kit (current version: SQK-LSK114), as described in the Methods section. This protocol usually requires 1.5 – 2 hours of hands-on time. It is most suitable for comprehensive whole genome sequencing aiming at sequencing a mammalian genome with approximately 30-fold coverage and an N50 of approximately 30kb. These parameters have recently been shown to be suitable for detecting individual disease-causing mutations for clinical human genetics on the PromethION platform with significantly higher throughput, albeit also substantially higher cost per flow cell [41, 42].

For routine QC of iPSC from non-model animals, we found the transposase-based rapid sequencing kit (current version: SQK-RAD114) on an inexpensive ONT MinION sequencer most useful, with less than 10 minutes of hands-on time for the library preparation protocol [39, 40]. This library preparation method generates smaller reads (5 – 15 kb), and a typical MinION flow cell can generate up to 3-4 million reads under ideal conditions.

4. Nanopore Sequencing:

We recommend nanopore sequencing for routine genomic QC of iPSC cultures aiming at about 1 million reads per run using the MinION platform. This parameter has been shown to enable the reliable detection of diagnostic CNVs and chromosomal amplifications and deletions in human cancers [43]. The sequencing library is loaded into the flow cell, and the sequencing run is started. Typically, sequencing times range for more than 1 million

reads on a MinION flow cell from 10 to 18 hours, depending on the number of available nanopores. The number of available nanopores will decrease as the sequencing progresses. A flow cell can be treated with a nuclease flush, washed, and additional libraries can be loaded sequentially to test multiple iPSC clones.

5. Bioinformatics:

While sequencing, online base calling can be performed with high-accuracy settings to convert raw signal data into nucleotide sequences. Refer to the manuscript for specific settings and considerations during nanopore sequencing. After obtaining sufficient nanopore reads, the generated reads are processed to assess genomic integrity.

The following steps are used in our prototypical genome integrity evaluation pipeline, designed specifically for this purpose. The code is available on Github at https://github.com/GJYWang/NWR_iPSC.git.

This pipeline integrates several bioinformatics tools and scripts that are used to align sequencing data to a high-quality reference genome, quantify copy number variations, and visualize genome integrity.

1. Dependencies

This section explains the software and tools that must be installed on the user's computer to run the bioinformatics pipeline. These tools, referred to as "dependencies," are essential for processing and analyzing the genomic data. Several specialized programs, such as minimap2 [13], samtools [44], and bedtools [45], are needed to handle the sequencing data. These programs help align the sequencing reads to a reference genome, manage the sequencing files, and analyze the data for any abnormalities in the genome. The user needs to install these tools and make sure they are correctly set up on their system. Sample code for installing these tools is available in the Github repository. The pipeline also requires specific packages for the R programming language, including DNAcopy [46] (https://github.com/veseshan/DNAcopy) and ggplot2. These packages are used for analyzing and visualizing the data, such as plotting graphs of genomic variations. Like the bioinformatics tools, these packages need to be installed in the R programming environment, and instructions for doing so can be found in the Github repository.

Specifically:

1.1 Bioinformatics tools including `minimap2`, `samtools`, and `bedtools` should be installed and included in the PATH. Sample code for installation can be found in the Github repository.

1.2 R packages including `DNAcopy` and `ggplot2` should be installed in R. Sample code for installation can be found in the Github repository.

1.3 The NWR reference genome can be downloaded from:
https://www.ncbi.nlm.nih.gov/datasets/genome/GCA_021442165.1/

Note: *The workflow is not specific to the NWR genome and can be used with any other similar chromosome-scale reference genome with a high contiguity.*

2. Usage

2.1 Please download the repository first from Github. The pipeline is implemented in file `pipeline.sh`.

2.2 Please modify the following variables in file `pipeline.sh`.

`assemblyPath`: Path to the assembly file downloaded from NCBI in 1.3. The NWR assembly file in fasta format. The fasta file needs to be indexed: `samtools faidx $assemblyPath`.

`fastq_file`: Path to the sequencing file in fastq.gz file format.

`alignment_path`: Path to store the intermediate alignment files.

`result_path`: Path to store the final results of genome integrity.

`thread`: Number of threads used for alignment.

`Bin_Size`: The size of the bin (in Mbps) for genome integrity analysis. We recommend to used bin size of 3Mbps.

2.3 After modifying file `pipeline.sh`. The pipeline can be run by command: `bash pipeline.sh`.

3. Output

All the results are stored in result_path. Four files will be generated:

3.1 `Read_Count.Rdata`: The read counts of each bin and the bin file is stored in Rdata format.

3.2 `bin_result.txt`: The copy number of each bin.

3.3 `segment.txt`: The segmented copy number results. This is the final genome integrity results to be used for visualization.

3.4 `genome_fig.pdf`: The visualization of genome integrity of the NWR lines.

Results such as those displayed in **Fig. S7** and **S8** will be generated by using this workflow. For **Fig. S8**, DNA was extracted from northern white rhinoceros (NWR) fibroblasts and an iPSC line, followed by ligation-based nanopore library preparation, as described in the Methods section. Two libraries, one per cell type, were sequenced on separate MinION nanopore flow cells. Each sequencing run was halted after approximately 12 hours, followed by nuclease flushing of the flow cell, and a second library was then loaded for sequencing. The results from each sequencing run, capturing the genomic integrity of both fibroblasts and iPSCs, are shown in **Fig. S8**.

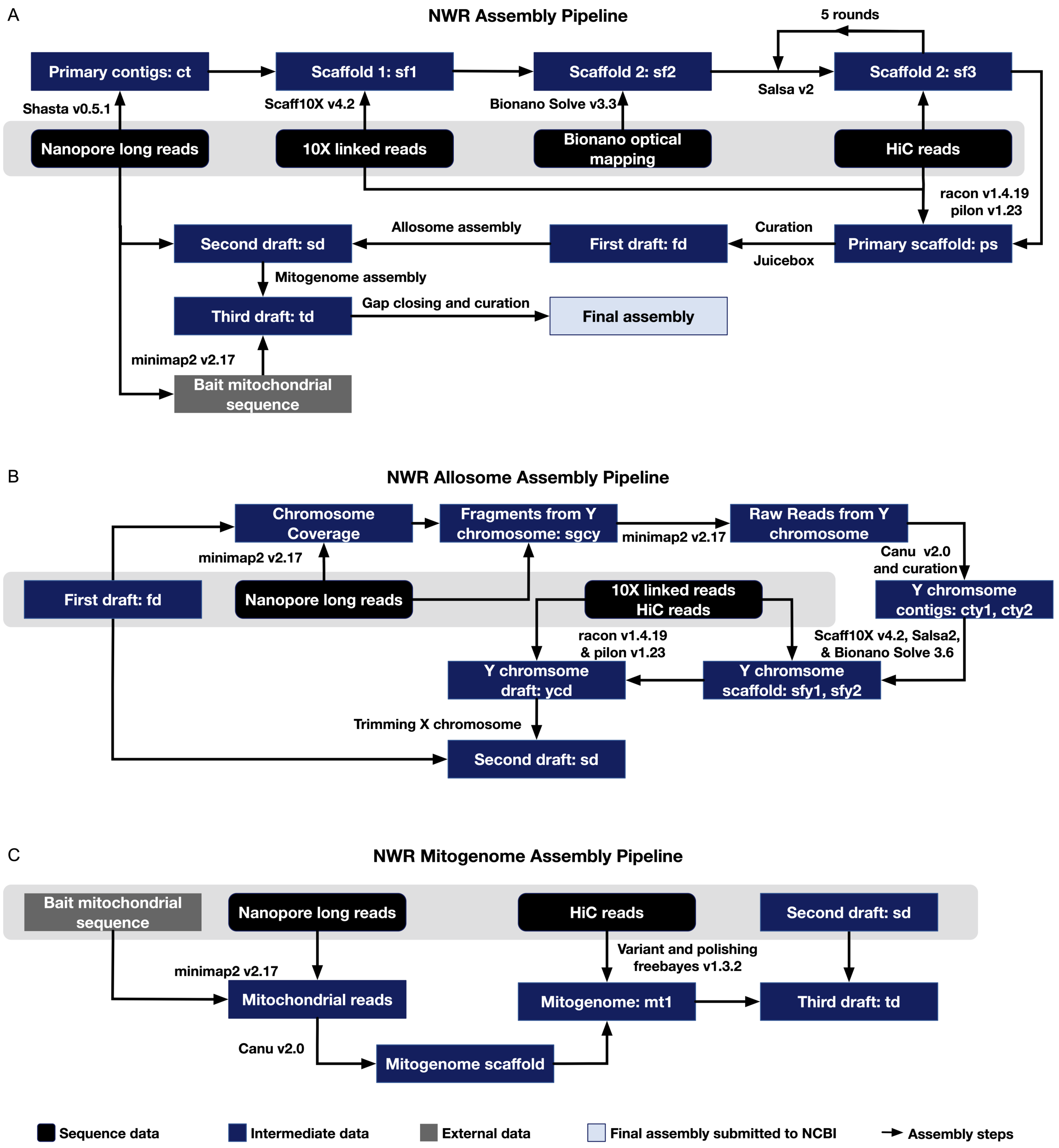

Fig. S1. Flow charts of the assembly pipelines used for the NWR genome. (a) Complete genome assembly pipeline used for the NWR reference genome: ONT long reads were assembled with Shasta into contigs. Scaffolds were generated with Scaff10x, were error corrected and scaffolded with Bionano Solve, and then sacffolded with 5 rounds of Salsa with HiC data. Scaffolded results were polished and curated to create the first draft assembly (fd): fd was then used for the allosome assembly pipeline to create the second draft (sd): sd was then used for the mitochondria assembly pipeline to generate the third draft. (b) Allosome assembly pipeline: Y chromosome reads were selected from the ONT reads by overlapping with DNA sequences of hybrid allosome in fd with coverage ranging from 56 and 81, the selected reads were assembled using Canu, scaffolded with Scaff10X, Salsa, and Bionano, then scaffolds were polished to generate the sd. (c) Mitochondrial genome assembly pipeline: bait mitochondrial sequence from the domestic horse was used to pull all mitochondrial reads from the ONT data; mitochondrial reads were then assembled using Canu; mitogenome (MT) was then polished and added to second draft to become the third draft.

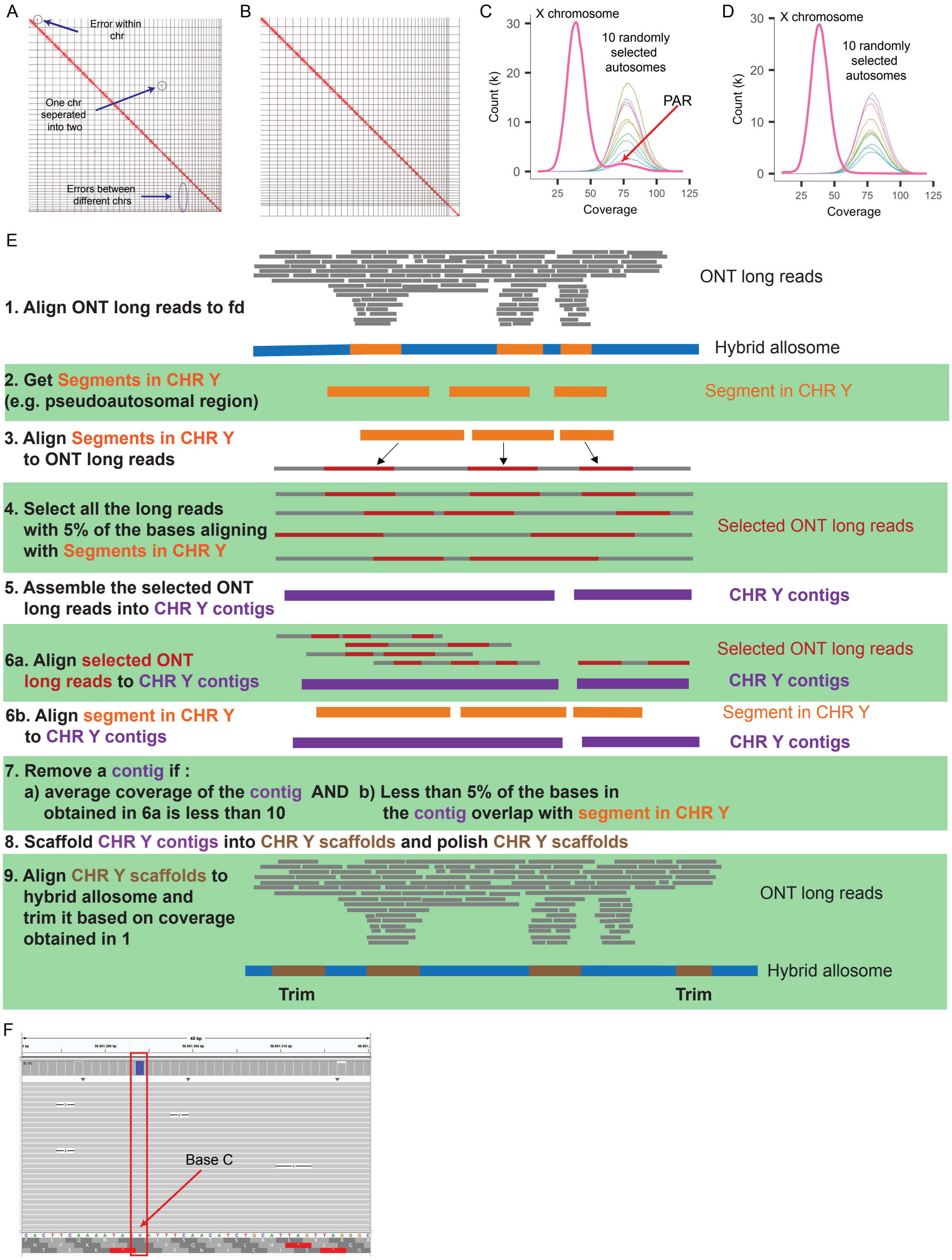

Fig.S2. Customized assembly process to improve the quality of the reference genome. (a) The HiC contact map before any manual curation. (b) The HiC contact map after manual curation using Juicebox. (c) The coverage histogram (randomly sampled from 10 Mbps) of the X chromosome and 10 randomly selected autosomes in first draft (fd) (d) The coverage histogram (randomly sampled from 10 Mbps) of the X chromosome and 10 randomly selected autosomes of CerSimCot1.0. (e) Detailed description of allosome assembly pipeline. (f) Using base calling consensus from ONT reads to close gaps.

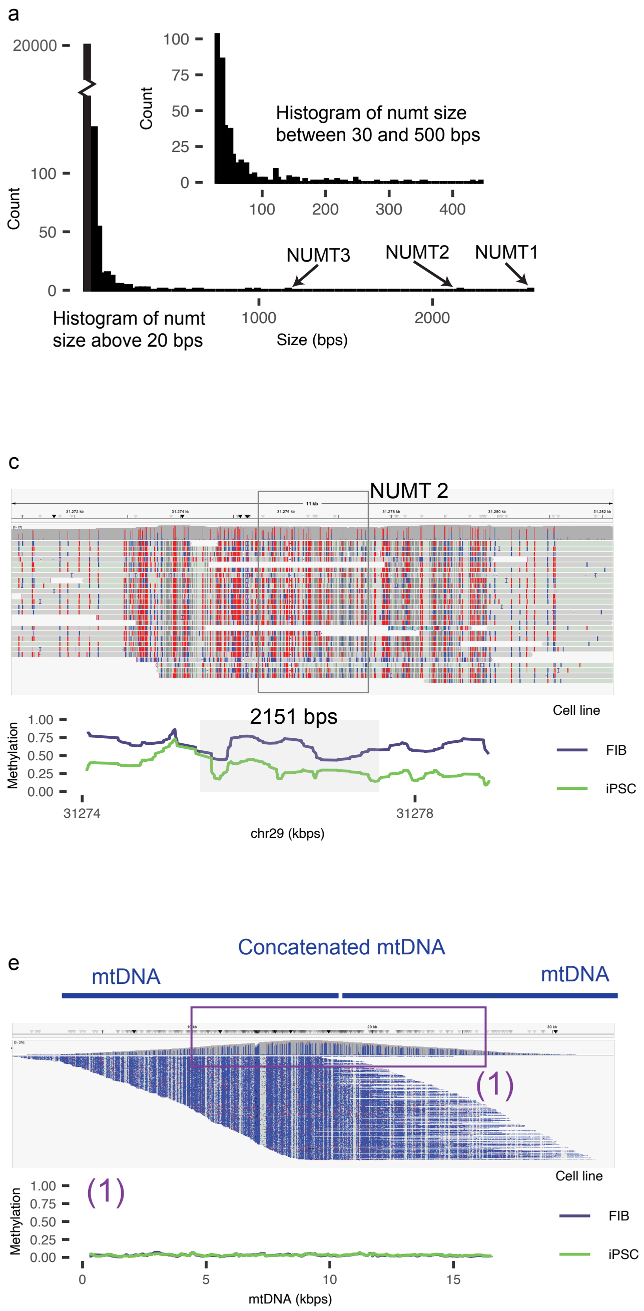

Fig. S3. NUMT mapping and CpG methylation analysis of NUMTs and the mitochondrial genome. (a) Size distribution of NUMTs. NUMTs smaller than 500 bp are shown in the inset. The three largest NUMTs are highlighted (b - d) DNA methylation analysis of the three largest NUMTs in their respective sequence context. Upper panel: read pileup in IGV. Lower panel: overall methylation rate in pileup. (e) DNA methylation analysis of reads used for the mitogenome assembly. Colors in read pileups (b-e): red: methylated CpG detected, blue: unmethylated CpG detected.

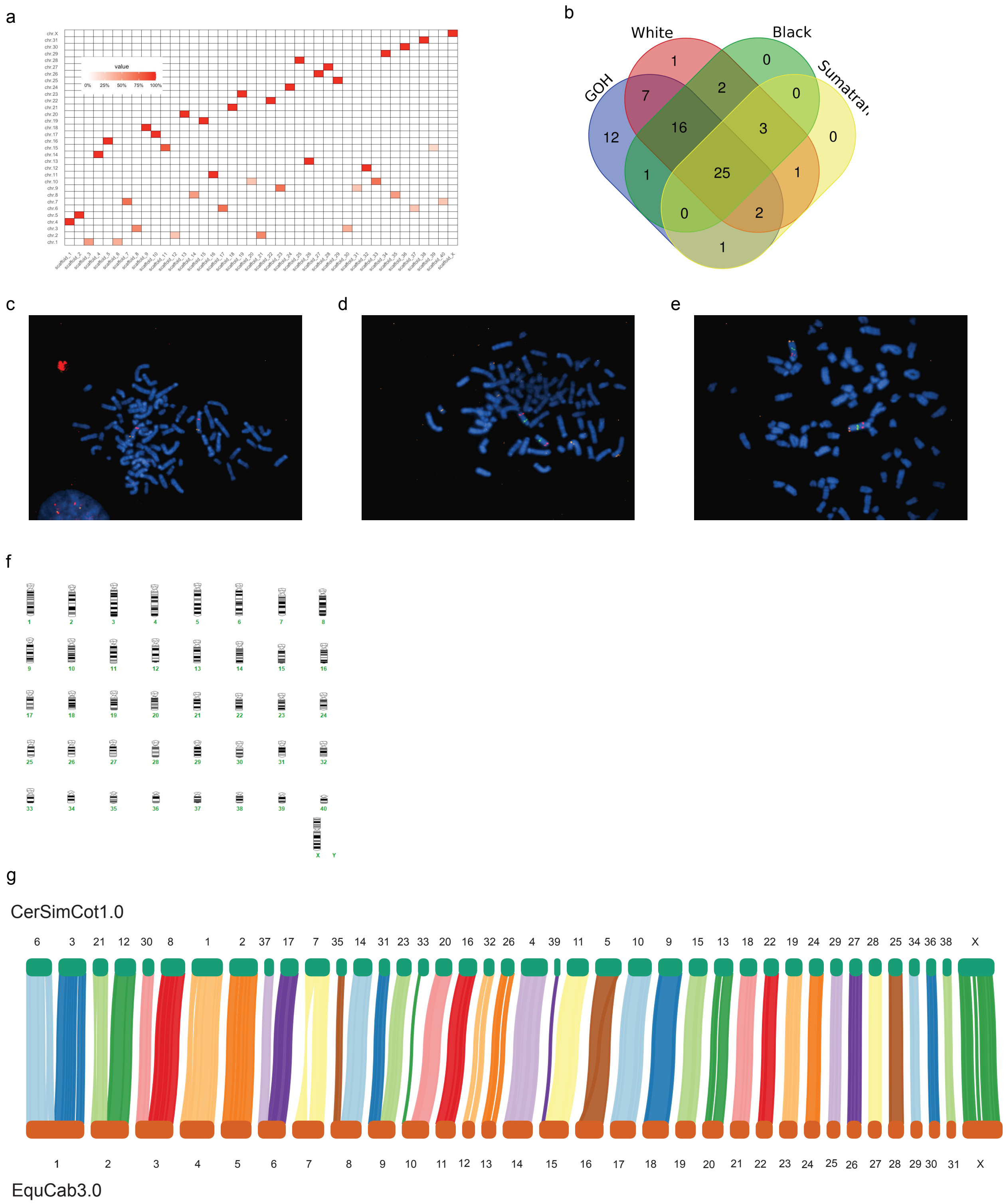

Fig.S4. Gene conservation with the horse and multiple rhinoceros species. (a) The percent of horse genes in each chromosome (Y axis) aligned to NWR chromosomes (X axis) is indicated. (b) Summary of bacterial artificial chromosomes (BACs) mapped across rhinoceros species, out of 71 total. (c-e) Representatives of FISH mapping experiments. (f) G-banded ideogram of all rhinoceros species with a 2n = 82 karyotype (Greater one-horned, Southern white, and Sumantran rhinoceros). (g) A synteny visualization created with NGenomeSyn based on alignment between CerSimCot1.0 and EquCab3.0.

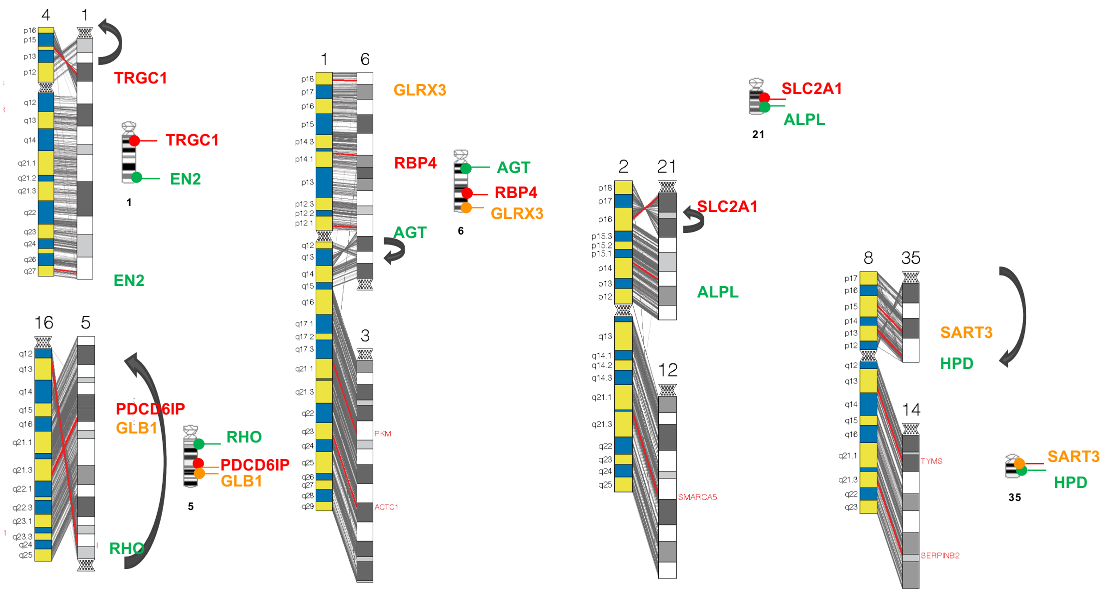

Fig. S5. Identification of assembly errors based on FISH mapping results. From left to right; TRGC1 should be located near the centromere on scaffold 1. Rho should be above PDCD6IP and GLB1 on scaffold 5. AGT should be closer to the centromere on scaffold 6. SLC2A1 should be further from the centromere on scaffold 21. SART3 should be closer to the centromere on scaffold 35.

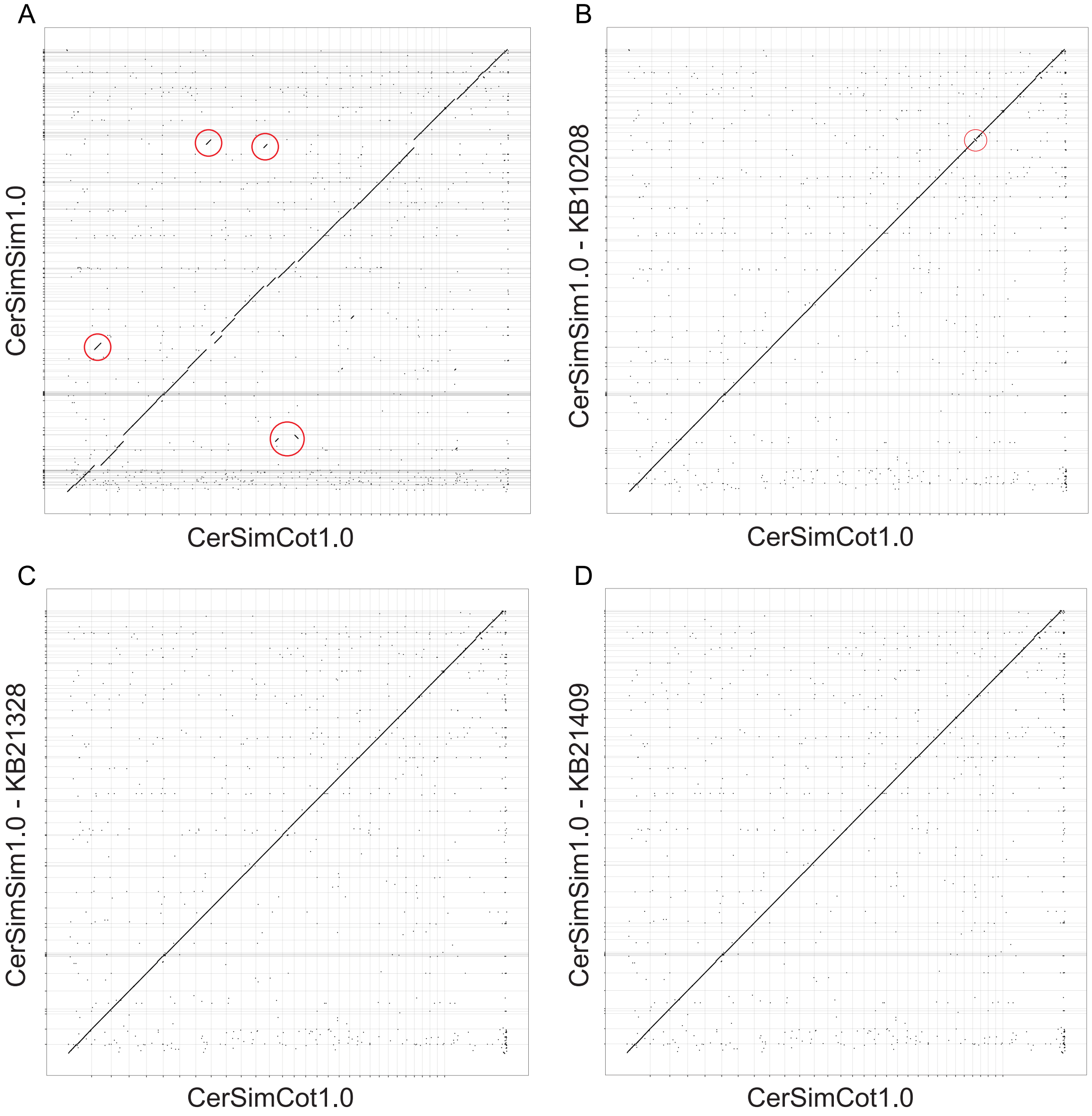

Fig. S6. Genome comparison between the NWR (CerSimCot1.0) and SWR genomes with dot-plots. (a) Comparison with the original SWR genome assembly (CerSimSim1.0). Potential assembly errors in CerSimSim1.0 are indicated by red circles. (b-d) Comparison with three additional SWR genomes (male KB10208 “Chuck”, female KB 21328 “Amani”, and female KB 21409 “Wallis”) with CerSimSim1.0 re-scaffolded using optical mapping. A small translocation identified in KB10208 is identified by a red circle.

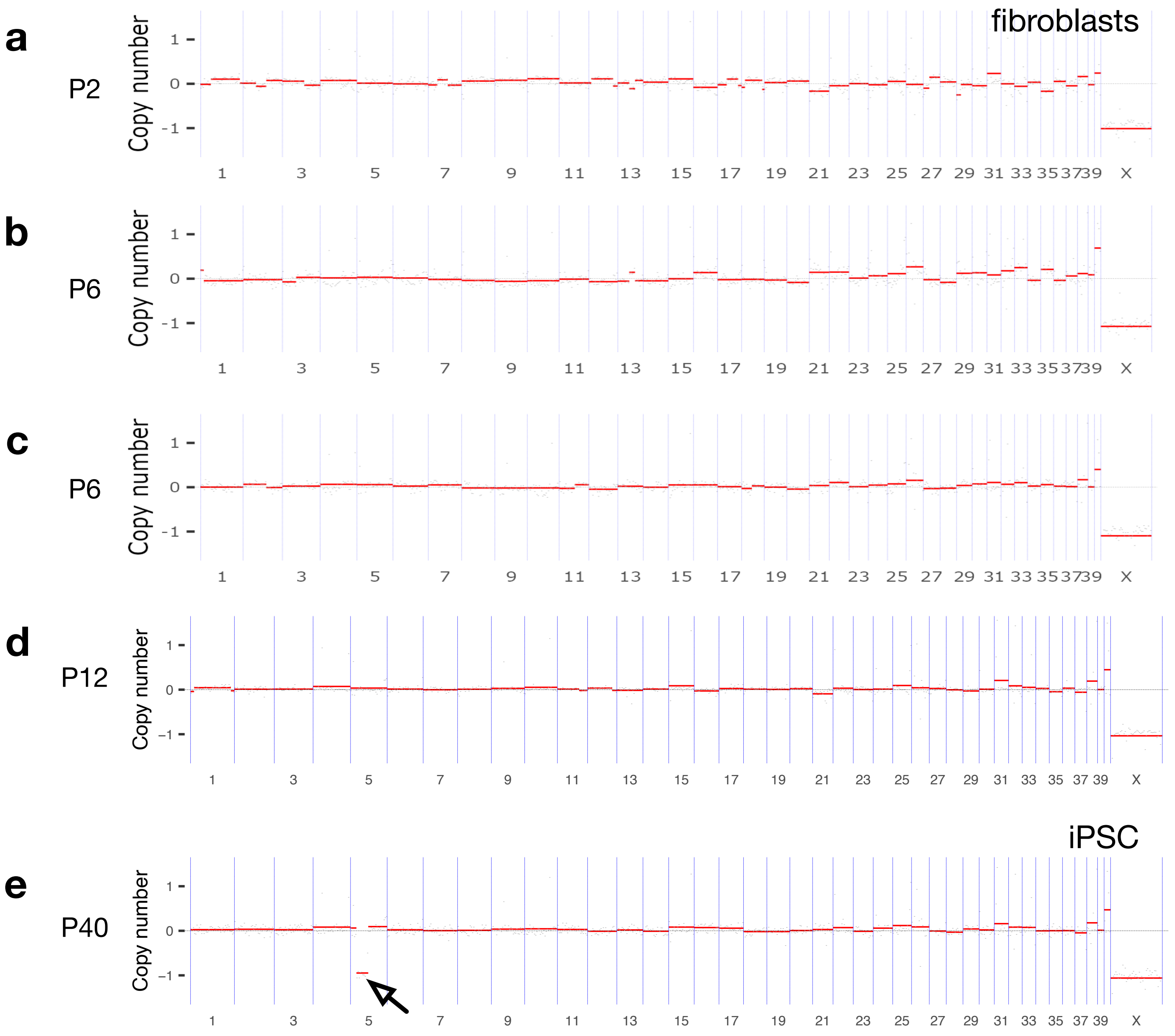

Fig. S7. Genomic integrity of NWR cell cultures. This graph shows bin values (small grey dots) on a log2 scale from fibroblast cultures P2 to 12 and iPSCs at passage 40 from the same NWR individual ("Angalifu", lab number KB9947). CNV analysis using Illumina short-reads and CerSimCot1.0 as reference genome. The red line represents a running average over the bin values. Copy number estimates are expressed in log2 ratio, where 0 represents diploidy and -1 represents haploidy. Only odd-numbered chromosomes are indicated in this visualization. All CNV profiles demonstrate hemizygosity for the X-chromosome in the male individual. (a - d) The copy number calls for fibroblast cultures from the same individual at different passages. (a) Illumina short read data at passage 2 [47] (b) short read data obtained with 10X Genomics linked reads at fibroblast passage 6. (c) short read data obtained with HiC sequencing at fibroblast passage 6. (c) long read data obtained with nanopore sequencing at fibroblast passage 12. (d) CNV of the iPSC line (NWR 9947-c501) at passage 40. The data displayed in (c) and (d) are identical to the data displayed in **Fig. 4**.

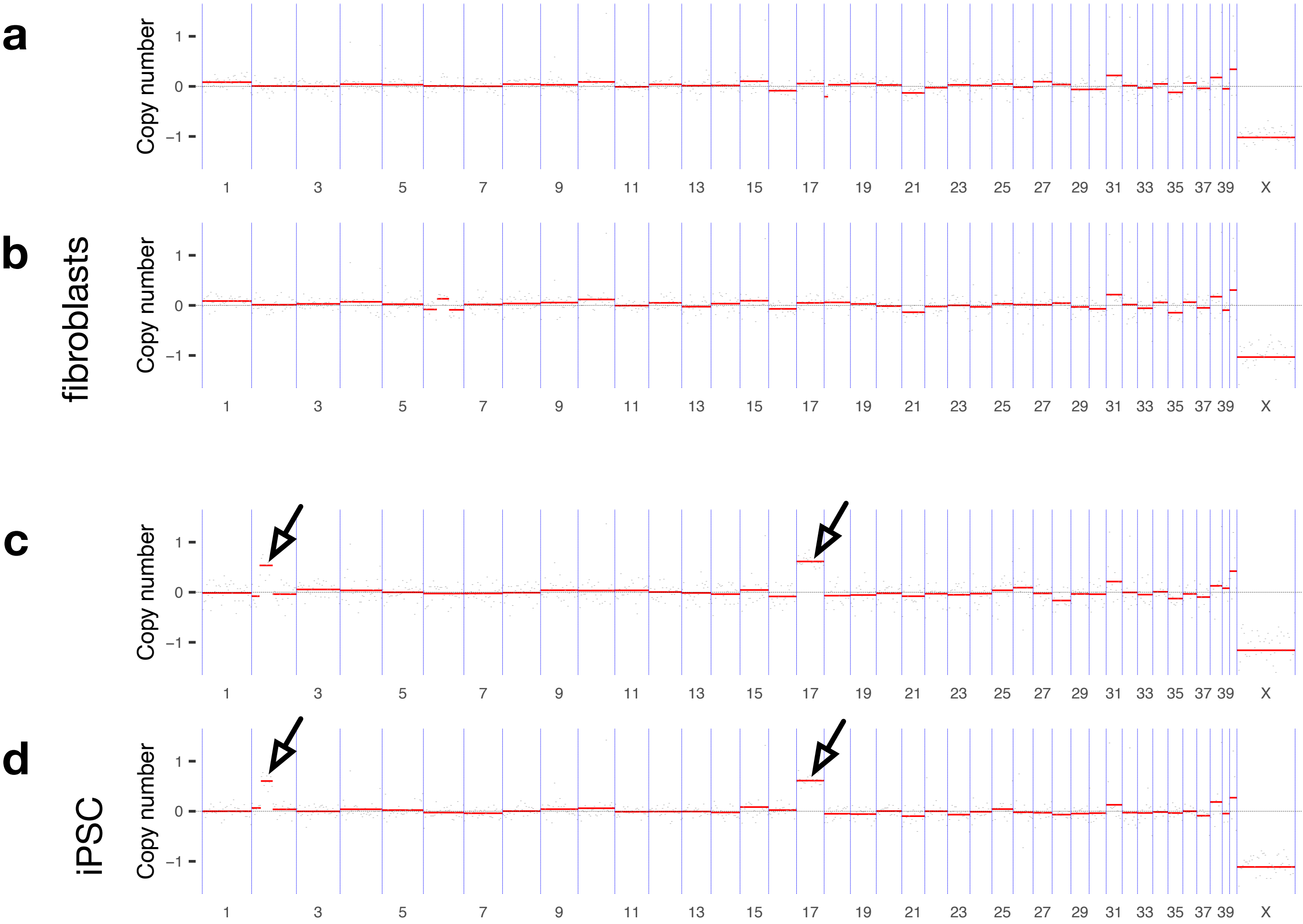

Fig. S8. Genomic integrity of fibroblasts and iPSC (9939-c5101) from another NWR individual ("Saut," laboratory number KB 9939). The same visualization principles are applied as in **Fig. S7**. Results shown are from one ONT flow cell per cell type (Fibroblasts FC#: FAN06288, iPSC FC#: FAN02719). After 12 hours, sequencing was stopped, a nuclease flush was conducted, and sequencing with a second library prepared from the same DNA source was restarted, increasing the overall sequencing yield. Data collected from each sequencing run is displayed (Fibroblasts: 26a27ac7 and 1de555bc, iPSC: cf7a00a9 and 7b2f97d4). All CNV profiles demonstrate hemizygosity for the X-chromosome in the male individual. Both iPSC runs show reproducible amplifications of a part of chromosome 2 and the whole chromosome 17 compared to results from the fibroblast cultures.

Table S1. Genome quality of NWR according to the proposed metrics of the Vertebrate Genome Project (part of the table comes from [21]).

Quality Category	Metric	Finished	VGP-2020	VGP-2016	VGP studies	NWR genome
**Notation**	x.y.P.Q.C	c.c.Pc.Q60.C100	7.c.P6.Q50.C95	7.c.P6.Q50.C95		
**Continuity**	Contig NG50 (x)	=Chr. NG50	>10 Mb	>10 Mb	1–25 Mb	**3.6 Mb**
	Scaffold NG50 (y)	=Chr. NG50	=Chr. NG50	=Chr. NG50	23–480 Mb	**Chr. NG50**
	Gaps per Gb	No gaps	<200	<200	75–1,500	**160**
**Structural accuracy**	Reliable blocks	=Chr. NG50	>10 Mb	>10 Mb	2.3–40.2 Mb	**11Mb**
	False duplications	0%	<1%	<1%	0.2–5.0%	**0.2%**
	Curation	Conflicts resolved	Manual	Manual	Manual	**Automated + Manual**
**Base accuracy**	Base pair QV (Q)	>60	>50	>50	39–43	**42**
	k-mer completeness	100% complete	>95%	>95%	87–98%	**92%**
**Haplotype phasing**	Phase block NG50 (P)	=Chr. NG50	>1 Mb	>1 Mb	1.6 Mb	**6.6 Mb**
**Functional completeness**	Genes	>98% complete	>95% complete	>95% complete	82–98%	**95.9% 92.0%**
	Transcript mappability	>98%	>90%	>90%	96%	**NA**
**Chromosome status**	Assigned (C)	>100%	>95%	>95%	94.4–99.9%	**100%**
	Sex chromosomes	Right order, no gaps	Localized homo pairs	Localized homo pairs	At least one shared	**Chr. X and partially resolved Y**
	Organelles (for example, MT)	One complete allele	One complete allele	One complete allele	One complete allele	**One complete allele**

Table S2. RNA-seq datasets used for annotation.	
**Individual**	**Cell or Tissue Type**
NWR KB9947	Fibroblasts
NWR KB9947	Feeder-dependent iPSCs
NWR KB9947, KB8173, KB8175, KB6571, KB17626	Embryoid bodies (14 days of differentiation)
NWR KB9947	Embryoid bodies (28 days of differentiation
NWR KB9947	Embryoid bodies (38 days of differentiation
NWR KB9947, KB8173, KB9939	Embryoid bodies (Day 2 suspension, Day 4 suspension, Day 7 suspension, Day 2 plated, Day 4 plated, Day 7 plated
NWR KB8173 and KB9939	Incipient mesoderm-like cells – 60 hours of differentiation
NWR KB8173	Primordial germ cell-like cells, Day 4 and Day 7 of differentiation
NWR KB6571	Partially reprogrammed iPSCs
NWR KB8175	Brain
NWR KB9947	Adult Testes
SWR KB21638	Neonate Testes
SWR NA	Mixed Female Reproductive Tissue

**Table S3.** Protein datasets used for annotation.		
**Common name**	**Scientific name**	**GenBank assembly accession**
Human	*Homo sapiens*	GCA_000001405.28
Mouse	*Mus musculus*	GCA_000001635.9
Southern white rhinoceros	*Ceratotherium simum simum*	GCA_000283155.1
Domestic horse	*Equus caballus*	GCA_002863925.1
Blue whale	*Balaenoptera musculus*	GCA_009873245.2

Table S4. List of bacterial artificial chromosome (BAC) clones used for fluorescent in situ hybridization. Clones are listed by gene name under which they were first published, with the current name used for mapping in bold. Clone identifier from either the CH241 (CHORI) or (INRA) BAC libraries. Original horse map locations and the predicted rhino chromosomes based on karyotype banding patterns are listed. GOHR = Great One-horned Rhino, SWR = southern white rhino, NWR = northern white rhino, SoBR = southern black rhino, EBR = eastern black rhino, SuR = Sumatran rhino. RSP6 and GNMT (italics) were removed from the final horse comparison because accurate sequence for these clones could not be found.

Gene Symbol	BAC clone ID	ECA Map Posi-tion	Ref.	Putative Rhino Chromo-some	NWR Scaf-fold	Probe Color	GOHR (ID)	SWR (ID)	NWR (ID)	SoBR (ID)	EBR (ID)	SuR(ID)
PICOT/TXNL2/**GLRX3**	INRA-0298E11	1p17	[48]	R6	6	SpO		Y (KB14068)	Y (KB5766)	Y (KB6944)	Y (KB10293)	Y (KB9200)
**RBP4**	INRA-386B12	1p14	[49]	R6	6	SpR	Y (KB13818)	Y (KB14068)	Y (KB5766)	Y (KB6944)	Y (KB10293)	Y (KB9200)
**AGT**	INRA-0296E05	1q12	[50]	R6	6	SpG	Y (KB13818)	Y (KB14068)	Y (KB5766)	Y (KB6944)	Y (KB10293)	Y (KB9200)
PKM2/**PKM**	INRA-399C9	1q21	[51]	R3	3	SpO	Y (KB13818)	Y (KB14068)	Y (KB5766)	Y (KB6944)	Y (KB10293)	Y (KB9200)
ACTC/**ACTC1**	INRA-208C9	1q24	[49]	R3	3	SpR	Y (KB13818) & (KB12915)	Y (KB14068)	Y (KB5766)	Y (KB6944)	Y (KB10293)	Y (KB9200)
**SLC2A1**	INRA-248F9	2p16	*	R20	21	SpR	Y (KB13818)	Y (KB8100)	Y (KB5766)		Y (KB10293)	
**ALPL**	INRA-20D8	2p14	[52]	R20	21	SpG	Y (KB13818)	Y (KB8100)	Y (KB5766)	Y (KB6944)	Y (KB10293)	
**SMARCA5**	INRA-281E7	2q21	[49, 53]	R13	12	SpO	Y (KB13818)		Y (KB5766)			
**KARS**	INRA-102B8	3p12-p13	[48, 49]	R9	30	SpR	Y (KB12915)	Y (KB13858)				
**MC1R**	INRA-104E10	3p12	[54-56]	R?	30	SpR	Y (KB14107) & (KB12797)					
**KIT**	INRA-258B12	3q21	[55, 57]	R9	8	SpO	Y (KB14107)					
**UCHL1**	INRA-208G12	3q22	[48, 49, 53, 58]	R9	8	SpG	Y (KB12915)					
**ADD1**	INRA-199C6	3q26	[48, 49]	R9	8	SpO	Y (KB12915)	Y (KB13858)				
TCRG/**TRGC1**	INRA-364A2	4p15	[49]	R2	1	SpR	Y (KB12915)	Y (KB13858)			Y (KB10293)	Y (KB9218)
**EN2**	INRA-175B2	4q27	[49]	R2	1	SpG	Y (KB12915)	Y (KB13858)			Y (KB10293)	Y (KB9218)
**LAMC2**	INRA-43H1	5p16-p17	[52]	R1	2	SpR	Y (KB12915)	Y (KB13858)	Y (KB9947)	Y (KB6944)	Y (KB10293)	Y (KB9218)

VDUP1/**TXNIP**	INRA-12G4	5p13	[49]	R1	2	SpG	Y (KB12915)	Y (KB13858)	Y (KB9947)	Y (KB6944)	Y (KB10293)	Y (KB9218)
**VCAM1**	INRA-53D7	5q14	[49]	R1	2	Biotin (Cy5)	Y (KB12915)					
DIA1/**CYB5R3**	INRA-5F2	5q17	[52]	R1	2	SpO	Y (KB12915)	Y (KB13858)	Y (KB9947)	Y (KB6944)	Y (KB10293)	Y (KB9218)
**INHA**	INRA-193E12	6p14	[52, 59, 60]	R38	37	SpO	Y (KB13818)		Y (KB5766)	Y (KB6944)		
**VWF**	INRA-34A12	6q12	[48, 54]	R19	17	SpR	Y (KB13818)		Y (KB5766)	Y (KB6944)	Y (KB10293)	
LY49L/**KLRB1**	CH241-303M11	6q22	[61]	R19	17	SpG	Y (KB13818)		Y (KB5766)	Y (KB6944)	Y (KB10293)	
**APOA4**	INRA-58E8	7p14-p15	[49]	R8	7	SpR	Y (KB12915)	Y (KB8100)	Y (KB9947)		Y (KB10293)	Y (KB9218)
**LYVE1**	INRA-344B1	7q17	[48]	R8	7	SpG	Y (KB12915)	Y (KB8100)	Y (KB9947)	Y (KB6944)	Y (KB10293)	Y (KB9218)
**HPD**	INRA-391C5	8p12-p13	[62]	R34	35	SpG	Y (KB13818					
**SART3**	INRA-326B4	8p16-p15	[49]	R34	35	SpO	Y (KB13818)	Y (KB14068)	Y (KB5766)	Y (KB6430)	Y (KB10293)	
**TYMS**	INRA-35H2	8q12-q14	[51]	R15	14	SpG	Y (KB13818)	Y (KB14068)	Y (KB5766)	Y (KB6430)	Y (KB10293)	
PAI2/**SERPINB2**	INRA-289A2	8q21	[52]	R15	14	SpR	Y (KB13818)		Y (KB5766)			
SLUG/**SNAI2**	CH241-255O14	9p11-p12	[58]	R35	23	SpR	Y (KB14107)					
DORFIN/**RNF19A**	CH241-1270N02	9q14	[58]	R35	23	SpG	Y (KB14107)					
AAT10/**SLC7A10**	INRA-638E6	10p15	[63]	R33	33	SpR	Y (KB12915)	Y (KB8100)	Y (KB9947)	Y (KB6944)	Y (KB10293)	Y (KB9218)
**ERCC2**	INRA-395A2	10p12	[52]	R33	33	SpG	Y (KB12915)	Y (KB8100)	Y (KB9947)	Y (KB6944)	Y (KB10293)	Y (KB9218)
**ELOVL4**	INRA-864H11	10q13	[58]	R17	20	SpG	Y (KB12797)	Y (KB14068)				
**PREP**	CH241-338C04	10q17	[58]	R17	20	SpR	Y (KB12797)	Y (KB14068)			Y (KB10293)	
**REV3L**	CH241-030KO7	10q21	[58]	R17	20	SpO	Y (KB12797)	Y (KB14068)			Y (KB10293)	
**P4HB**	CH241-331I4	11p14	[64]	R12	16	SpG			Y (KB9947)	Y (KB6430)		
**GH1**	CH241-477J2	11q14	[65]	R12	16	SpR	Y (KB12915)		Y (KB9947)	Y (KB6430)		
**OMG**	INRA-39B3	11q14	[48, 49]	R12	16	SpO		Y (KB14068)		Y (KB6430)		
**TRAF6**	INRA-058J14	12p13	[58]	R31	32	SpO	Y (KB13818)	Y (KB14068)		Y (KB6430)		
CD20/**MS4A1**	CH241-190J19	12q13	[48, 64, 66]	R31	32	SpG	Y (KB13818)	Y (KB8100)		Y (KB6944)		Y (KB9200)
ADRBK1/**GRK2**	CH241-362F21	12q14	[64]	R31	32	SpR	Y (KB13818)	Y (KB8100)		Y (KB6944)		Y (KB9200)

**POR**	INRA-330G11	13p13	[62]	R23	26	SpO	Y (KB12915)	Y (KB13858)	Y (KB9947)	Y (KB6944)	Y (KB10293)	Y (KB9218)
**GUSB**	CH241-110C9	13q11	[64]	R23	26	SpG	Y (KB12915)	Y (KB13858)	Y (KB9947)	Y (KB6944)	Y (KB10293)	Y (KB9218)
MHCG5/**KIF3A**	CH241-441E15	14q21	[61]	R5	4	SpO	Y (KB13818)					
**SPTBN1**	INRA-21G9	15q22	[51, 67]	R11	11	SpR	Y (KB12915)	Y (KB13858)	Y (KB9947)	Y (KB6944)	Y (KB10293)	Y (KB9218)
**APOB**	INRA-128E12	15q25-q26	[62]	R11	11	SpG	Y (KB12915)	Y (KB13858)	Y (KB9947)	Y (KB6944)	Y (KB10293)	Y (KB9218)
**RHO**	CH241-452O13	16q11	[48, 64]	R4	5	SpG	Y (KB10301)	Y (KB8100)	Y (KB5766)	Y (KB13606)	Y (KB10293)	Y (KB9200)
**PDCD6IP**	INRA-0089G8	16q21	[48]	R4	5	SpR	Y (KB10301)	Y (KB8100)	Y (KB5766)	Y (KB13606)	Y (KB10293)	
**GLB1**	INRA-144N5	16q22	[64]	R4	5	SpO	Y (KB10301)	Y (KB8100)	Y (KB5766)	Y (KB13606)	Y (KB10293)	Y (KB9200)
**MTMR6**	INRA-225D8	17q13	[48]	R10	10	SpO	Y (KB10301)	Y (KB8100)	Y (KB5766)		Y (KB10293)	
**ALOX5AP**	INRA-170C8	17q14-q15	[52, 62]	R10	10	SpG	Y (KB10301)	Y (KB8100)	Y (KB5766)		Y (KB10293)	
**EDNRB**	INRA-152D3	17q23-q24	[62]	R10	10	SpR		Y (KB8100)	Y (KB5766)			
**CXCR4**	INRA-186C10	18q15	[48]	R16	9	SpR	Y (KB12915)	Y (KB14068)	Y (KB5766)			
**CHRNA1**	CH241-470F16	18q25	[48, 64]	R16	9	SpO	Y (KB12915)	Y (KB14068)	Y (KB5766)			Y (KB9218)
RPS6	*INRA-0326E11*	*19q14*	*[48]*	*R7*	*15*	*SpO*		Y (KB14068)				
**MHC**	CH241-288J19	20q21	[68]	R14	13	SpR	Y (KB13818)	Y (KB8100)	Y (KB5766)	Y (KB13606)	Y (KB10293)	
**ITPR3**	INRA-66C7	20q21.1	[49]	R14	13	SpG	Y (KB13818)	Y (KB8100)	Y (KB5766)	Y (KB13606)	Y (KB10293)	
CTLA3/**GZMA**	CH241-151J1	21q13	[48]	R21	18	SpO	Y (KB13818)		Y (KB5766)	Y (KB13606)	Y (KB10293)	
**GNAS**	INRA-105L2	22q18-q19	[48, 64]	R25	22	SpR	Y (KB12915)					Y (KB9218)
**TGFB3**	INRA-31D2	24q14	[62]	R22	24	SpO	Y (KB12797)					
GNMT	*INRA-228G6*	*24q16*		*R22*	*24*	*SpO/SpR*	Y (KB12797	Y (KB14068)				
GRP78/**HSPA5**	CH241-185K3	25q18-q19	[64]	R30	29	SpR		Y (KB14068)	Y (KB5766)		Y (KB10293)	Y (KB9200)
**GBE1**	CH241-14K06	26q12	[69]	R27	27	SpG	Y (KB14107)	Y (KB14068)				
DSCR1/**RCAN1**	INRA-1124D05	26q15	[48]	R27	27	SpR	Y (KB14107)	Y (KB14068)				

**PDGFRL**	CH241-151K03	27q16	[58]	R24	28	SpO	Y (KB12797)					
MGF/**KITLG**	INRA-???	28q13	[70, 71]	R26	25	SpR	Y (KB9471)					
HESTG05/**NET1** (Goat BAC)	INRA-243H7	29q16	[54]	R36	34	SpR	Y (KB12915)	Y (KB8100)	Y (KB5766)		Y (KB10293)	Y (KB9218)
**TGFB2**	INRA-161H9	30q14	[62]	R29	36	SpO		Y (KB14068)				Y (KB9218)
**PLG**	INRA-313V12	31q12-q14	[48, 51]	R37	38	SpG	Y (KB12915)	Y (KB14068)	Y (KB5766)			Y (KB9218)
**OTC**	INRA-58G6	Xp15-p16	[54]	RXp	X	SpO	Y (KB13818)	Y (KB8100)	Y (KB5766)	Y (KB13606)	Y (KB10293)	Y (KB9200)
TRAP170/**MED14**	INRA-14E8	Xp14-p13/p16-p14??	[72]	RXp	X	SpR	Y (KB12915)	Y (KB14068)	Y (KB5766)	Y (KB13606)	Y (KB10293)	Y (KB9200)
**PGK1**	INRA-860H5	Xq13	[62]	RXq	X	SpO		Y (KB14068)	Y (KB5766)	Y (KB13606)	Y (KB10293)	Y (KB9200)
**GPC3**	INRA-180E6	Xq27	[49]	RXq	X	SpG	Y (KB12915)	Y (KB14068)	Y (KB5766)	Y (KB13606)	Y (KB10293)	Y (KB9200)

* This paper

Table S5. Individual information for the rhino fibroblasts used for slide making and FISH hybridizations.

Slides Used for Hybridizations	KB #	Sex	Accession #	Location	DOB	DOD	SB#	2n	House Name
Greater One-horned Rhino (*Rhinoceros unicornis*)	13818	M	601279	SD-WAP	01-Nov-89 (est.)	29-Oct-20	190	82	Arun
	12915	M	601332	SD-WAP	6-Jun-01	1-Feb-05	277	82	Gyan
	10301	M	698678	SD-WAP	21-Dec-98	21-Dec-98	258	82	N/A
	12797	M	699439	SD-WAP	27-Jul-99	Undetermined	262	82	Jiibito
	14107	M	699277	SD-WAP	18-May-99	Undetermined	259	82	Gram
	9471	F	102575	SD-WAP	19-Mar-78	15-Dec-04	99	82	Gainda
Southern White Rhino (Ceratotherium simum simum)	14068	F	695051	SD-WAP	26-Feb-95	13-Jan-04	1051	82	Ujima
	13858	F	601441	SD-WAP	19-Aug-01	alive	1391	82	Kibibi
	8100	F	026342	SD-WAP	28-Aug-84	29-Jun-02	822	82	Sinyaa
Northern White Rhino (*Ceratotherium simum cottoni*)	5766	M	059001	DVURKRALV	01-Jan-73 (est.)	19-Mar-18	372	81	Sudan
	9947	M	690500	SD-WAP	01-Apr-72 (est.)	14-Dec-14	348	82	Angalifu
Southern Black Rhino (*Diceros bicornis bicornis*)	6430	M	587408	SANDIEGOZ	15-May-86 (est.)	9-Jan-04	390	83	Gundwane, Little
	6944	F	589278	SANDIEGOZ	18-Mar-86 (est.)	16-Apr-20	392	84	Chirundu-Mimi
	13606	M	597233	SANDIEGOZ	31-May-97	18-Apr-19	681	83	Limpopo
Eastern Black Rhino (*Diceros bicornis michaeli*)	10293	M	696516	SD-WAP	6-Oct-96	alive	636	83	Jubba
Sumatran Rhino (*Dicerorhinus sumatrensis*)	9200	F	588440	SANDIEGOZ	24-Jul-88 (est.)	22-Feb-95	25	81	Barakas-Kumu
	9218	M	592372	SANDIEGOZ	20-Mar-92 (est.)	27-Feb-95	35	82	Tanjung

Table S6. Genome quality of NWR and SWR assemblies according to the proposed metrics of the Vertebrate Genome Project (part of the table comes from [21]).

Quality Category	Metric	CerSimCot1.0	CerSimSim 1.0	CerSimSim1.0 - KB10208	CerSimSim1.0 – KB21328	CerSimSim1.0 - KB21409
**Individual**		**"Angalifu" (KB9947)**	**SDZICR (KB13650)**	**"Chuck" (KB10208)**	**"Amani" (KB21328)**	**"Wallis" (KB21409)**
**Year finished**		**2024**	2012	**2024**	**2024**	**2024**
**Continuity**	Contig NG50 (x)	**3.6 Mb**	93 kb	93 kb	93 kb	93 kb
	Scaffold NG50 (y)	**Chr. NG50**	26.3 Mb	63Mb	63Mb	62Mb
	Number of scaffolds	**41**	3087	66	69	64
	Gaps per Gb	**160**	22948	22699	22782	22500
**Structural accuracy**	Reliable blocks	**11Mb**	NA	NA	NA	NA
	False duplications	**0.2%**	NA	NA	NA	NA
	Curation	**Automated + Manual**	NA	NA	NA	NA
**Haplotype phasing**	Phase block NG50 (P)	**6.6 Mb**	NA	NA	NA	NA
**Chromo-some status**	Assigned (C)	**100%**	NA	91%	100%	100%
	Sex chromosomes	**Chr. X and partially resolved Y**	partially resolved X	partially resolved X	partially resolved X	partially resolved X
	Organelles (for example, MT)	MT complete	MT complete	MT complete	MT complete	MT complete

Comparison between the Northern White Rhino assembly (CerSimCot1.0), the Southern White Rhino assembly (CerSimSim1.0), and CerSimSim1.0 scaffolded with optical maps from three SWR individuals. The highest achieved metrics values are highlighted in **bold.**

Table S7. Summary of successful BAC hybridizations by species.

Greater One-Horned Rhinoceros	Southern White Rhinoceros	Northern White Rhinoceros	Southern Black Rhinoceros	Eastern Black Rhinoceros	Sumatran Rhinoceros
64	51	45	38	40	32

Table S8. Annotated genes heterozygously deleted in NWR iPSC line at passage 40.
Blue: Known tumor suppressor genes; Red: Genes involved in various stages of meiosis.

GENE SYMBOL	GENE NAME	ENSEMBL ID
**AADAT**	aminoadipate aminotransferase(AADAT)	ENSG00000109576
**ABCB10**	ATP binding cassette subfamily B member 10(ABCB10)	ENSG00000135776
**ACAD11**	acyl-CoA dehydrogenase family member 11(ACAD11)	ENSG00000240303
**ACAD8**	acyl-CoA dehydrogenase family member 8(ACAD8)	ENSG00000151498
**ACAT1**	acetyl-CoA acetyltransferase 1(ACAT1)	ENSG00000075239
**ACKR4**	atypical chemokine receptor 4(ACKR4)	ENSG00000129048
**ADARB1**	adenosine deaminase RNA specific B1(ADARB1)	ENSG00000197381
**ADCY2**	adenylate cyclase 2(ADCY2)	ENSG00000078295
**ADCYAP1**	adenylate cyclase activating polypeptide 1(ADCYAP1)	ENSG00000141433
**ADIPOR1**	adiponectin receptor 1(ADIPOR1)	ENSG00000159346
**AGTR1**	angiotensin II receptor type 1(AGTR1)	ENSG00000144891
**AK1**	adenylate kinase 1(AK1)	ENSG00000106992
**ALMS1**	ALMS1 centrosome and basal body associated protein(ALMS1)	ENSG00000116127
**AMOTL2**	angiomotin like 2(AMOTL2)	ENSG00000114019
**ANAPC13**	anaphase promoting complex subunit 13(ANAPC13)	ENSG00000129055
**ANKRD28**	ankyrin repeat domain 28(ANKRD28)	ENSG00000206560
**ANKRD36**	ankyrin repeat domain 36(ANKRD36)	ENSG00000135976
**AOC2**	amine oxidase copper containing 2(AOC2)	ENSG00000131480
**ARHGEF26**	Rho guanine nucleotide exchange factor 26(ARHGEF26)	ENSG00000114790
**ARMC8**	armadillo repeat containing 8(ARMC8)	ENSG00000114098
**ASB1**	ankyrin repeat and SOCS box containing 1(ASB1)	ENSG00000065802
**ATP1B3**	ATPase Na+/K+ transporting subunit beta 3(ATP1B3)	ENSG00000069849
**ATP2C1**	ATPase secretory pathway Ca2+ transporting 1(ATP2C1)	ENSG00000017260
**ATP6V1A**	ATPase H+ transporting V1 subunit A(ATP6V1A)	ENSG00000114573
**ATR**	ATR serine/threonine kinase(ATR)	ENSG00000175054
**BFSP2**	beaded filament structural protein 2(BFSP2)	ENSG00000170819
**BTD**	biotinidase(BTD)	ENSG00000169814
**C3ORF33**	chromosome 3 open reading frame 33(C3orf33)	ENSG00000174928
**C8ORF31**	chromosome 8 open reading frame 31	ENSG00000177335
**CACNA1S**	calcium voltage-gated channel subunit alpha1 S(CACNA1S)	ENSG00000081248
**CAPN1**	calpain 1(CAPN1)	ENSG00000014216
**CASC16**	cancer susceptibility 16(CASC16)	ENSG00000249231
**CCNB3**	cyclin B3(CCNB3)	ENSG00000147082
**CDV3**	CDV3 homolog(CDV3)	ENSG00000091527
**CENPJ**	centromere protein J(CENPJ)	ENSG00000151849
**CENPM**	centromere protein M(CENPM)	ENSG00000100162
**CEP63**	centrosomal protein 63(CEP63)	ENSG00000182923
**CEP70**	centrosomal protein 70(CEP70)	ENSG00000114107
**CHST2**	carbohydrate sulfotransferase 2(CHST2)	ENSG00000175040
**CLDN18**	claudin 18(CLDN18)	ENSG00000066405

**CLRN1**	clarin 1(CLRN1)	ENSG00000163646
**CLSTN2**	calsyntenin 2(CLSTN2)	ENSG00000158258
**CNTN1**	contactin 1(CNTN1)	ENSG00000018236
**CNTRL**	centriolin(CNTRL)	ENSG00000119397
**COL4A6**	collagen type IV alpha 6 chain(COL4A6)	ENSG00000197565
**COL6A5**	collagen type VI alpha 5 chain(COL6A5)	ENSG00000172752
**COL6A6**	collagen type VI alpha 6 chain(COL6A6)	ENSG00000206384
**COLQ**	collagen like tail subunit of asymmetric acetylcholinesterase(COLQ)	ENSG00000206561
**COMMD2**	COMM domain containing 2(COMMD2)	ENSG00000114744
**COPB2**	COPI coat complex subunit beta 2(COPB2)	ENSG00000184432
**COX8A**	cytochrome c oxidase subunit 8A(COX8A)	ENSG00000176340
**CP**	ceruloplasmin(CP)	ENSG00000047457
**CPA3**	carboxypeptidase A3(CPA3)	ENSG00000163751
**CPD**	carboxypeptidase D(CPD)	ENSG00000108582
**CPNE4**	copine 4(CPNE4)	ENSG00000196353
**CRABP2**	cellular retinoic acid binding protein 2(CRABP2)	ENSG00000143320
**DAPK2**	death associated protein kinase 2(DAPK2)	ENSG00000035664
**DAZL**	deleted in azoospermia like(DAZL)	ENSG00000092345
**DBR1**	debranching RNA lariats 1(DBR1)	ENSG00000138231
**DHX36**	DEAH-box helicase 36(DHX36)	ENSG00000174953
**DNAJC13**	DnaJ heat shock protein family (Hsp40) member C13(DNAJC13)	ENSG00000138246
**DPH3**	diphthamide biosynthesis 3(DPH3)	ENSG00000154813
**DZIP1L**	DAZ interacting zinc finger protein 1 like(DZIP1L)	ENSG00000158163
**E2F2**	E2F transcription factor 2(E2F2)	ENSG00000007968
**EAF1**	ELL associated factor 1(EAF1)	ENSG00000144597
**EFHB**	EF-hand domain family member B(EFHB)	ENSG00000163576
**EIF2A**	eukaryotic translation initiation factor 2A(EIF2A)	ENSG00000144895
**EIF4B**	eukaryotic translation initiation factor 4B(EIF4B)	ENSG00000063046
**EMCN**	endomucin(EMCN)	ENSG00000164035
**EPHB1**	EPH receptor B1(EPHB1)	ENSG00000154928
**ERAL1**	Era like 12S mitochondrial rRNA chaperone 1(ERAL1)	ENSG00000132591
**ERI1**	exoribonuclease 1(ERI1)	ENSG00000104626
**ESYT3**	extended synaptotagmin 3(ESYT3)	ENSG00000158220
**EYA2**	EYA transcriptional coactivator and phosphatase 2(EYA2)	ENSG00000064655
**FAIM**	Fas apoptotic inhibitory molecule(FAIM)	ENSG00000158234
**FBXW5**	F-box and WD repeat domain containing 5(FBXW5)	ENSG00000159069
**FIGLA**	folliculogenesis specific bHLH transcription factor(FIGLA)	ENSG00000183733
**FKBP8**	FKBP prolyl isomerase 8(FKBP8)	ENSG00000105701
**FLNA**	filamin A(FLNA)	ENSG00000196924
**FOXL2**	forkhead box L2(FOXL2)	ENSG00000183770
**FXYD4**	FXYD domain containing ion transport regulator 4(FXYD4)	ENSG00000150201
**GALNT15**	polypeptide N-acetylgalactosaminyltransferase 15(GALNT15)	ENSG00000131386
**GPR149**	G protein-coupled receptor 149(GPR149)	ENSG00000174948

**GPR87**	G protein-coupled receptor 87(GPR87)	ENSG00000138271
**GRK7**	G protein-coupled receptor kinase 7(GRK7)	ENSG00000114124
**GYG1**	glycogenin 1(GYG1)	ENSG00000163754
**HACE1**	HECT domain and ankyrin repeat containing E3 ubiquitin protein ligase 1(HACE1)	ENSG00000085382
**HACL1**	2-hydroxyacyl-CoA lyase 1(HACL1)	ENSG00000131373
**HERC3**	HECT and RLD domain containing E3 ubiquitin protein ligase 3(HERC3)	ENSG00000138641
**HES3**	hes family bHLH transcription factor 3(HES3)	ENSG00000173673
**HLA-DQA1**	major histocompatibility complex, class II, DQ alpha 1(HLA-DQA1)	ENSG00000196735
**HLA-DQB1**	major histocompatibility complex, class II, DQ beta 1(HLA-DQB1)	ENSG00000179344
**HLA-DRB1**	major histocompatibility complex, class II, DR beta 1(HLA-DRB1)	ENSG00000196126
**HLTF**	helicase like transcription factor(HLTF)	ENSG00000071794
**HPS3**	HPS3 biogenesis of lysosomal organelles complex 2 subunit 1(HPS3)	ENSG00000163755
**HSPA8**	heat shock protein family A (Hsp70) member 8(HSPA8)	ENSG00000109971
**IFNL2**	interferon lambda 2(IFNL2)	ENSG00000183709
**IGSF10**	immunoglobulin superfamily member 10(IGSF10)	ENSG00000152580
**IL1RAP**	interleukin 1 receptor accessory protein(IL1RAP)	ENSG00000196083
**IL20RB**	interleukin 20 receptor subunit beta(IL20RB)	ENSG00000174564
**INS**	insulin(INS)	ENSG00000254647
**KAT2B**	lysine acetyltransferase 2B(KAT2B)	ENSG00000114166
**KCNH8**	potassium voltage-gated channel subfamily H member 8(KCNH8)	ENSG00000183960
**KDM1B**	lysine demethylase 1B(KDM1B)	ENSG00000165097
**KIAA0753**	KIAA0753(KIAA0753)	ENSG00000198920
**KLF15**	KLF transcription factor 15(KLF15)	ENSG00000163884
**KLHL36**	kelch like family member 36(KLHL36)	ENSG00000135686
**KY**	kyphoscoliosis peptidase(KY)	ENSG00000174611
**LVRN**	laeverin(LVRN)	ENSG00000172901
**LYPD4**	LY6/PLAUR domain containing 4(LYPD4)	ENSG00000273111
**MADD**	MAP kinase activating death domain(MADD)	ENSG00000110514
**MAGI2**	membrane associated guanylate kinase, WW and PDZ domain containing 2(MAGI2)	ENSG00000187391
**MBNL1**	muscleblind like splicing regulator 1(MBNL1)	ENSG00000152601
**MDM1**	Mdm1 nuclear protein(MDM1)	ENSG00000111554
**METTL6**	Methyltransferase 6, TRNA N3-Cytidine	ENSG00000206562
**MINDY4B**	MINDY family member 4B(MINDY4B)	ENSG00000214237
**MME**	membrane metalloendopeptidase(MME)	ENSG00000196549
**MSL2**	MSL complex subunit 2(MSL2)	ENSG00000174579
**MYH4**	myosin heavy chain 4(MYH4)	ENSG00000264424
**NCK1**	NCK adaptor protein 1(NCK1)	ENSG00000158092
**NEK11**	NIMA related kinase 11(NEK11)	ENSG00000114670
**NEK9**	NIMA related kinase 9(NEK9)	ENSG00000119638

**NKIRAS1**	NFKB inhibitor interacting Ras like 1(NKIRAS1)	ENSG00000197885
**NLRP3**	NLR family pyrin domain containing 3(NLRP3)	ENSG00000162711
**NLRP5**	NLR family pyrin domain containing 5(NLRP5)	ENSG00000171487
**NME9**	NME/NM23 family member 9(NME9)	ENSG00000181322
**NMNAT3**	nicotinamide nucleotide adenylyltransferase 3(NMNAT3)	ENSG00000163864
**NPHP3**	nephrocystin 3(NPHP3)	ENSG00000113971
**NSUN2**	NOP2/Sun RNA methyltransferase 2(NSUN2)	ENSG00000037474
**NUDT16**	nudix hydrolase 16(NUDT16)	ENSG00000198585
**NYAP2**	neuronal tyrosine-phosphorylated phosphoinositide-3-kinase adaptor 2(NYAP2)	ENSG00000144460
**OR2B2**	olfactory receptor family 2 subfamily B member 2(OR2B2)	ENSG00000168131
**OR2F1**	olfactory receptor family 2 subfamily F member 1(OR2F1)	ENSG00000213215
**OVOS2**	alpha-2-macroglobulin like 1 pseudogene(OVOS2)	ENSG00000177359
**OXNAD1**	oxidoreductase NAD binding domain containing 1(OXNAD1)	ENSG00000154814
**P2RY1**	purinergic receptor P2Y1(P2RY1)	ENSG00000169860
**P2RY12**	purinergic receptor P2Y12(P2RY12)	ENSG00000169313
**P2RY13**	purinergic receptor P2Y13(P2RY13)	ENSG00000181631
**P2RY14**	purinergic receptor P2Y14(P2RY14)	ENSG00000174944
**P2RY6**	pyrimidinergic receptor P2Y6(P2RY6)	ENSG00000171631
**PAQR9**	progestin and adipoQ receptor family member 9(PAQR9)	ENSG00000188582
**PCCB**	propionyl-CoA carboxylase subunit beta(PCCB)	ENSG00000114054
**PCDH18**	protocadherin 18(PCDH18)	ENSG00000189184
**PFKFB1**	6-phosphofructo-2-kinase/fructose-2,6-biphosphatase 1(PFKFB1)	ENSG00000158571
**PFN2**	profilin 2(PFN2)	ENSG00000070087
**PIK3CB**	phosphatidylinositol-4,5-bisphosphate 3-kinase catalytic subunit beta(PIK3CB)	ENSG00000051382
**PIK3R4**	phosphoinositide-3-kinase regulatory subunit 4(PIK3R4)	ENSG00000196455
**PITPNM2**	phosphatidylinositol transfer protein membrane associated 2(PITPNM2)	ENSG00000090975
**PLCH1**	phospholipase C eta 1(PLCH1)	ENSG00000114805
**PLCL2**	phospholipase C like 2(PLCL2)	ENSG00000154822
**PLOD2**	procollagen-lysine,2-oxoglutarate 5-dioxygenase 2(PLOD2)	ENSG00000152952
**PLPP1**	phospholipid phosphatase 1(PLPP1)	ENSG00000067113
**PLPP2**	phospholipid phosphatase 2(PLPP2)	ENSG00000141934
**PLS1**	plastin 1(PLS1)	ENSG00000120756
**PLSCR4**	phospholipid scramblase 4(PLSCR4)	ENSG00000114698
**PLSCR5**	phospholipid scramblase family member 5(PLSCR5)	ENSG00000231213
**PPOX**	protoporphyrinogen oxidase(PPOX)	ENSG00000143224
**PPP2R1A**	protein phosphatase 2 scaffold subunit Aalpha(PPP2R1A)	ENSG00000105568
**PRR23A**	proline rich 23A(PRR23A)	ENSG00000206260
**PSME3IP1**	proteasome activator subunit 3 interacting protein 1(PSME3IP1)	ENSG00000172775
**PTDSS2**	phosphatidylserine synthase 2(PTDSS2)	ENSG00000174915
**PXYLP1**	2-phosphoxylose phosphatase 1(PXYLP1)	ENSG00000155893
**RAB5A**	RAB5A, member RAS oncogene family(RAB5A)	ENSG00000144566

**RAB6B**	RAB6B, member RAS oncogene family(RAB6B)	ENSG00000154917
**RAP2B**	RAP2B, member of RAS oncogene family(RAP2B)	ENSG00000181467
**RASA2**	RAS p21 protein activator 2(RASA2)	ENSG00000155903
**RASGRF1**	Ras protein specific guanine nucleotide releasing factor 1(RASGRF1)	ENSG00000058335
**RBP1**	retinol binding protein 1(RBP1)	ENSG00000114115
**RET**	ret proto-oncogene (RET)	ENSG00000165731
**RFTN1**	raftlin, lipid raft linker 1(RFTN1)	ENSG00000131378
**RFX1**	regulatory factor X1(RFX1)	ENSG00000132005
**RMDN3**	regulator of microtubule dynamics 3(RMDN3)	ENSG00000137824
**RNF13**	ring finger protein 13(RNF13)	ENSG00000082996
**RNF7**	ring finger protein 7(RNF7)	ENSG00000114125
**RPL15**	ribosomal protein L15(RPL15)	ENSG00000174748
**RPSA**	ribosomal protein SA(RPSA)	ENSG00000168028
**RTTN**	rotatin(RTTN)	ENSG00000176225
**RYK**	receptor like tyrosine kinase(RYK)	ENSG00000163785
**SAP18**	Sin3A associated protein 18(SAP18)	ENSG00000150459
**SATB1**	SATB homeobox 1(SATB1)	ENSG00000182568
**SELENOT**	selenoprotein T(SELENOT)	ENSG00000198843
**SEMA4F**	ssemaphorin 4F(SEMA4F)	ENSG00000135622
**SERP1**	stress associated endoplasmic reticulum protein 1(SERP1)	ENSG00000120742
**SGO1**	shugoshin 1(SGO1)	ENSG00000129810
**SH3BP5**	SH3 domain binding protein 5(SH3BP5)	ENSG00000131370
**SHC2**	SHC adaptor protein 2(SHC2)	ENSG00000129946
**SIAH2**	siah E3 ubiquitin protein ligase 2(SIAH2)	ENSG00000181788
**SLC25A36**	solute carrier family 25 member 36(SLC25A36)	ENSG00000114120
**SLC29A4**	solute carrier family 29 member 4(SLC29A4)	ENSG00000164638
**SLC35G2**	solute carrier family 35 member G2(SLC35G2)	ENSG00000168917
**SLC5A1**	solute carrier family 5 member 1(SLC5A1)	ENSG00000100170
**SLC9A9**	solute carrier family 9 member A9(SLC9A9)	ENSG00000181804
**SLCO2A1**	solute carrier organic anion transporter family member 2A1(SLCO2A1)	ENSG00000174640
**SNRK**	SNF related kinase(SNRK)	ENSG00000163788
**SNRNP200**	small nuclear ribonucleoprotein U5 subunit 200(SNRNP200)	ENSG00000144028
**SNX20**	sorting nexin 20(SNX20)	ENSG00000167208
**SOAT1**	sterol O-acyltransferase 1(SOAT1)	ENSG00000057252
**SOX14**	SRY-box transcription factor 14(SOX14)	ENSG00000168875
**SPSB4**	splA/ryanodine receptor domain and SOCS box containing 4(SPSB4)	ENSG00000175093
**SRPRB**	SRP receptor subunit beta(SRPRB)	ENSG00000144867
**STAG1**	STAG1 cohesin complex component(STAG1)	ENSG00000118007
**STK36**	serine/threonine kinase 36(STK36)	ENSG00000163482
**TAF1D**	TATA-box binding protein associated factor, RNA polymerase I subunit D(TAF1D)	ENSG00000166012
**TBC1D5**	TBC1 domain family member 5(TBC1D5)	ENSG00000131374
**TF**	transferrin(TF)	ENSG00000091513

**TFDP2**	transcription factor Dp-2(TFDP2)	ENSG00000114126
**TMEM108**	transmembrane protein 108(TMEM108)	ENSG00000144868
**TOPBP1**	DNA topoisomerase II binding protein 1(TOPBP1)	ENSG00000163781
**TRGC1**	T cell receptor gamma constant 1(TRGC1)	ENSG00000211689
**TRIM28**	tripartite motif containing 28(TRIM28)	ENSG00000130726
**TRIM42**	tripartite motif containing 42(TRIM42)	ENSG00000155890
**TRIML1**	tripartite motif family like 1(TRIML1)	ENSG00000184108
**TRPC1**	transient receptor potential cation channel subfamily C member 1(TRPC1)	ENSG00000144935
**TXNIP**	thioredoxin interacting protein(TXNIP)	ENSG00000265972
**TXNRD1**	thioredoxin reductase 1(TXNRD1)	ENSG00000198431
**UBA5**	ubiquitin like modifier activating enzyme 5(UBA5)	ENSG00000081307
**UBE2E1**	ubiquitin conjugating enzyme E2 E1(UBE2E1)	ENSG00000170142
**UBE2E2**	ubiquitin conjugating enzyme E2 E2(UBE2E2)	ENSG00000182247
**WEE2**	WEE2 oocyte meiosis inhibiting kinase(WEE2)	ENSG00000214102
**WWTR1**	WW domain containing transcription regulator 1(WWTR1)	ENSG00000018408
**XKR6**	XK related 6(XKR6)	ENSG00000171044
**XRN1**	5'-3' exoribonuclease 1(XRN1)	ENSG00000114127
**ZBTB38**	zinc finger and BTB domain containing 38(ZBTB38)	ENSG00000177311
**ZC3H8**	zinc finger CCCH-type containing 8(ZC3H8)	ENSG00000144161
**ZIC1**	Zic family member 1(ZIC1)	ENSG00000152977
**ZIC4**	Zic family member 4(ZIC4)	ENSG00000174963
**ZNF132**	zinc finger protein 132(ZNF132)	ENSG00000131849
**ZNF263**	zinc finger protein 263(ZNF263)	ENSG00000006194
**ZNF385B**	zinc finger protein 385B(ZNF385B)	ENSG00000144331
**ZNF385D**	zinc finger protein 385D(ZNF385D)	ENSG00000151789
**ZP3**	zona pellucida glycoprotein 3(ZP3)	ENSG00000188372

Seven genes that play important roles in human gamete development and function:

DAZL (Deleted in Azoospermia-Like): This gene is relevant for germ cell development and meiosis. It is involved in the regulation of translation in germ cells and is essential for spermatogenesis and oogenesis [73, 74].

STAG1 (Stromal Antigen 1: Part of the cohesin complex, which is important for the proper segregation of chromosomes during meiosis [75].

ANAPC13 (Anaphase Promoting Complex Subunit 13): This gene is part of the anaphase-promoting complex/cyclosome (APC/C), a regulator of the cell cycle, including the progression of cells through meiosis [76].

EYA2 (EYA Transcriptional Coactivator and Phosphatase 2): Playes a role in the development and maintenance of various tissues, including germ cells. It regulates in DNA repair processes, which are involved in meiosis [74].

SGO1 (Shugoshin 1): Plays a key role in protecting centromeric cohesion during meiosis, ensuring accurate chromosome segregation [77].

TOPBP1 (DNA Topoisomerase II Binding Protein 1): Essential for DNA replication and repair, processes that are critical for maintaining genome stability during meiosis [78].

ZP3 (Zona Pellucida Glycoprotein 3): While primarily involved in sperm binding and induction of the acrosome reaction during fertilization, its role is critical for the interaction of gametes.

Two oncogenes with a role in cancer development when heterozygously deleted:

1. ATR (ATR serine/threonine kinase): Heterozygous mutations or deletions of ATR can compromise its function, leading to defects in DNA repair and replication processes [79].
2. ZIC1 (Zic family member 1): Downregulation of ZIC1 is associated with the development and progression of gastric cancers [80].

SI References

1. Joseph, S. and W. David, *Preparation and analysis of eukaryotic genomic DNA.* Molecular Cloning: A Laboratory Manual, 2001. **1**: p. 6.1-6.64.
2. Shafin, K., et al., *Nanopore sequencing and the Shasta toolkit enable efficient de novo assembly of eleven human genomes.* Nature Biotechnology, 2020. **38**(9): p. 1044-1053.
3. Ning, Z., F. Giordano, and E. Harry. *Scaffold10X*. 2019; Available from: https://github.com/wtsi-hpag/Scaff10X.
4. Inc., A.G. *Arima Genomics Mapping pipeline*. Available from: https://github.com/ArimaGenomics/mapping_pipeline.
5. Li, H., *Aligning sequence reads, clone sequences and assembly contigs with BWA-MEM.* arXiv: Genomics, 2013.
6. Institute, B. *Picard Tools*. 2018; Available from: http://broadinstitute.github.io/picard/.
7. Ghurye, J., et al., *Integrating Hi-C links with assembly graphs for chromosome-scale assembly.* PLOS Computational Biology, 2019. **15**(8): p. e1007273.
8. Vaser, R., et al., *Fast and accurate de novo genome assembly from long uncorrected reads.* Genome Research, 2017.
9. Walker, B.J., et al., *Pilon: An Integrated Tool for Comprehensive Microbial Variant Detection and Genome Assembly Improvement.* PLOS ONE, 2014. **9**(11): p. e112963.
10. Durand, N.C., et al., *Juicer Provides a One-Click System for Analyzing Loop-Resolution Hi-C Experiments.* Cell Systems, 2016. **3**(1): p. 95-98.
11. Dudchenko, O., et al., *De novo assembly of the Aedes aegypti genome using Hi-C yields chromosome-length scaffolds.* Science, 2017. **356**(6333): p. 92-95.
12. Robinson, J.T., et al., *Juicebox.js Provides a Cloud-Based Visualization System for Hi-C Data.* Cell Systems, 2018. **6**(2): p. 256-258.e1.
13. Li, H., *Minimap2: pairwise alignment for nucleotide sequences.* Bioinformatics, 2018. **34**(18): p. 3094-3100.
14. Koren, S., et al., *Canu: scalable and accurate long-read assembly via adaptive k-mer weighting and repeat separation.* Genome Research, 2017. **27**(5): p. 722-736.
15. Kalbfleisch, T.S., et al., *Improved reference genome for the domestic horse increases assembly contiguity and composition.* Communications Biology, 2018. **1**(1): p. 197.
16. Formenti, G., et al., *Complete vertebrate mitogenomes reveal widespread repeats and gene duplications.* Genome Biology, 2021. **22**(1): p. 120.
17. Edwards, R.J., et al., *Chromosome-length genome assembly and structural variations of the primal Basenji dog (Canis lupus familiaris) genome.* BMC Genomics, 2021. **22**(1): p. 188.
18. Robinson, J.T., et al., *Integrative genomics viewer.* Nature Biotechnology, 2011. **29**(1): p. 24-26.
19. Rhie, A., et al., *Merqury: reference-free quality, completeness, and phasing assessment for genome assemblies.* Genome Biology, 2020. **21**(1): p. 245.
20. Ranallo-Benavidez, T.R., K.S. Jaron, and M.C. Schatz, *GenomeScope 2.0 and Smudgeplot for reference-free profiling of polyploid genomes.* Nature Communications, 2020. **11**(1): p. 1432.
21. Rhie, A., et al., *Towards complete and error-free genome assemblies of all vertebrate species.* Nature, 2021. **592**(7856): p. 737-746.
22. Garrison, E. and G. Marth, *Haplotype-based variant detection from short-read sequencing.* arXiv preprint arXiv:1207.3907, 2012.
23. Martin, M., et al., *WhatsHap: fast and accurate read-based phasing.* bioRxiv, 2016: p. 085050.
24. Simão, F.A., et al., *BUSCO: assessing genome assembly and annotation completeness with single-copy orthologs.* Bioinformatics, 2015. **31**(19): p. 3210-3212.
25. Podlevsky, J.D., et al., *The Telomerase Database.* Nucleic Acids Research, 2007. **36**(suppl_1): p. D339-D343.
26. Ruby, J.G., P. Bellare, and J.L. DeRisi, *PRICE: Software for the Targeted Assembly of Components of (Meta) Genomic Sequence Data.* G3 Genes|Genomes|Genetics, 2013. **3**(5): p. 865-880.

27. Benson, G., *Tandem repeats finder: a program to analyze DNA sequences.* Nucleic Acids Research, 1999. **27**(2): p. 573-580.
28. Smit, A., R. Hubley, and P. Green. *RepeatMasker Open-4.0*. 2013-2015; Available from: http://www.repeatmasker.org.
29. Dobin, A., et al., *STAR: ultrafast universal RNA-seq aligner.* Bioinformatics, 2013. **29**(1): p. 15-21.
30. Lars, G., et al., *BRAKER3: Fully Automated Genome Annotation Using RNA-Seq and Protein Evidence with GeneMark-ETP, AUGUSTUS and TSEBRA.* bioRxiv, 2023: p. 2023.06.10.544449.
31. Consortium, T.U., *UniProt: the universal protein knowledgebase in 2021.* Nucleic Acids Research, 2020. **49**(D1): p. D480-D489.
32. He, W., et al., *NGenomeSyn: an easy-to-use and flexible tool for publication-ready visualization of syntenic relationships across multiple genomes.* Bioinformatics, 2023. **39**(3).
33. Poorten, T. *dotPlotly*. 2018; Available from: https://github.com/tpoorten/dotPlotly.dotPlotly.
34. Ben-Nun, I.F., et al., *Induced pluripotent stem cells from highly endangered species.* Nat Methods, 2011. **8**(10): p. 829-31.
35. Korody, M.L., et al., *Rewinding Extinction in the Northern White Rhinoceros: Genetically Diverse Induced Pluripotent Stem Cell Bank for Genetic Rescue.* Stem Cells and Development, 2021. **30**(4): p. 177-189.
36. Hildebrandt, T.B., et al., *Embryos and embryonic stem cells from the white rhinoceros.* Nature Communications, 2018. **9**(1): p. 2589.
37. Hayashi, M., et al., *Robust induction of primordial germ cells of white rhinoceros on the brink of extinction.* Science Advances, 2022. **8**(49): p. eabp9683.
38. Zywitza, V., et al., *Naïve-like pluripotency to pave the way for saving the northern white rhinoceros from extinction.* Scientific Reports, 2022. **12**(1): p. 3100.
39. Djirackor, L., et al., *Intraoperative DNA methylation classification of brain tumors impacts neurosurgical strategy.* Neurooncol Adv, 2021. **3**(1): p. vdab149.
40. Vermeulen, C., et al., *Ultra-fast deep-learned CNS tumour classification during surgery.* Nature, 2023. **622**(7984): p. 842-849.
41. Kolmogorov, M., et al., *Scalable Nanopore sequencing of human genomes provides a comprehensive view of haplotype-resolved variation and methylation.* Nature Methods, 2023. **20**(10): p. 1483-1492.
42. Negi, S., et al., *Advancing long-read nanopore genome assembly and accurate variant calling for rare disease detection.* medRxiv, 2024: p. 2024.08.22.24312327.
43. Thongrattana, W., et al., *GLIMMERS: glioma molecular markers exploration using long-read sequencing.* Bioinformatics Advances, 2024. **4**(1).
44. Li, H., et al., *The Sequence Alignment/Map format and SAMtools.* Bioinformatics, 2009. **25**(16): p. 2078-2079.
45. Quinlan, A.R. and I.M. Hall, *BEDTools: a flexible suite of utilities for comparing genomic features.* Bioinformatics, 2010. **26**(6): p. 841-842.
46. Olshen, A.B., et al., *Circular binary segmentation for the analysis of array‐based DNA copy number data.* Biostatistics, 2004. **5**(4): p. 557-572.
47. Tunstall, T., et al., *Evaluating recovery potential of the northern white rhinoceros from cryopreserved somatic cells.* Genome Research, 2018.
48. Chowdhary, B.P., et al., *The first-generation whole-genome radiation hybrid map in the horse identifies conserved segments in human and mouse genomes Erratum: Genome Res. 13, 1258 (2003).* Genome Res, 2003. **13**(4): p. 742-51.
49. Lear, T.L., et al., *Mapping of 31 horse genes in BACs by FISH.* Chromosome Res, 2001. **9**(3): p. 261-2.
50. Raudsepp, T., et al., *A 4,103 marker integrated physical and comparative map of the horse genome.* Cytogenet Genome Res, 2008. **122**(1): p. 28-36.
51. Lear, T.L., et al., *Horse v-fes feline sarcoma viral oncogene homologue; pyruvate kinase, muscle type 2; plasminogen; beta spectrin, non-erythrocytic 1; thymidylate synthetase; and microsatellite LEX078 map to 1q14-q15, 1q21, 31q12-q14, 15q22, 8q12-q14, and 14q27, respectively.* Chromosome Res, 2000. **8**(4): p. 361.

52. Mariat, D., et al., *Isolation, characterization and FISH assignments of horse BAC clones containing type I and II markers.* Cytogenet Cell Genet, 2001. **92**(1-2): p. 144-8.
53. Myka, J.L., et al., *Homologous fission event(s) implicated for chromosomal polymorphisms among five species in the genus Equus.* Cytogenet Genome Res, 2003. **102**(1-4): p. 217-21.
54. Godard, S., et al., *Cytogenetic localization of 44 new coding sequences in the horse.* Mamm Genome, 2000. **11**(12): p. 1093-7.
55. Raudsepp, T., et al., *Comparison of horse chromosome 3 with donkey and human chromosomes by cross-species painting and heterologous FISH mapping.* Mamm Genome, 1999. **10**(3): p. 277-82.
56. Wagner, H.J. and M. Reissmann, *New polymorphism detected in the horse MC1R gene.* Anim Genet, 2000. **31**(4): p. 289-90.
57. Lear, T.L., L.D. Coogle, and E. Bailey, *Assignment of the horse mitochondrial glutamate oxaloacetate transaminase 2 (GOT2) and v-kit Hardy-Zuckerman 4 feline sarcoma viral oncogene homolog (KIT) to horse chromosome 3 by in situ hybridization.* Cytogenet Cell Genet, 1998. **82**(1-2): p. 112-3.
58. Perrocheau, M., et al., *Construction of a medium-density horse gene map.* Anim Genet, 2006. **37**(2): p. 145-55.
59. Murphy, B.A., et al., *Chromosomal assignments and sequences for the equine core circadian clock genes.* Anim Genet, 2007. **38**(1): p. 84-5.
60. Myka, J.L., et al., *FISH analysis comparing genome organization in the domestic horse (Equus caballus) to that of the Mongolian wild horse (E. przewalskii).* Cytogenet Genome Res, 2003. **102**(1-4): p. 222-5.
61. Leeb, T., et al., *A human-horse comparative map based on equine BAC end sequences.* Genomics, 2006. **87**(6): p. 772-6.
62. Milenkovic, D., et al., *Cytogenetic localization of 136 genes in the horse: comparative mapping with the human genome.* Mamm Genome, 2002. **13**(9): p. 524-34.
63. Hanzawa, K., et al., *Mapping of equine potassium chloride co-transporter (SLC12A4) and amino acid transporter (SLC7A10) and preliminary studies on associations between SNPs from SLC12A4, SLC7A10 and SLC7A9 and osmotic fragility of erythrocytes.* Anim Genet, 2002. **33**(6): p. 455-9.
64. Caetano, A.R., et al., *A comparative gene map of the horse (Equus caballus).* Genome Res, 1999. **9**(12): p. 1239-49.
65. Caetano, A.R., et al., *Comparative mapping of 18 equine type I genes assigned by somatic cell hybrid analysis.* Mamm Genome, 1999. **10**(3): p. 271-6.
66. Musilova, P., et al., *Cytogenetic mapping of immunity-related genes in the domestic horse.* Anim Genet, 2005. **36**(6): p. 507-10.
67. Wagner, M.L., et al., *A 1.3-Mb interval map of equine homologs of HSA2.* Cytogenet Genome Res, 2006. **112**(3-4): p. 227-34.
68. Mains, C.M., *Comparative Mapping: Homology Within the Order Perissodactyla of Four Genes Located on Equus Caballus Chromosome 20*, in *Veterinary Science*. 2004, University of Kentucky.
69. Ward, T.L., et al., *Genetic mapping of GBE1 and its association with glycogen storage disease IV in American Quarter horses.* Cytogenet Genome Res, 2003. **102**(1-4): p. 201-6.
70. Marklund, S., et al., *Close association between sequence polymorphism in the KIT gene and the roan coat color in horses.* Mamm Genome, 1999. **10**(3): p. 283-8.
71. Terry, R.B., et al., *Rejection of MITF and MGF as the genes responsible for appaloosa coat colour patterns in horses.* Anim Genet, 2002. **33**(1): p. 82-4.
72. Raudsepp, T., et al., *Conservation of gene order between horse and human X chromosomes as evidenced through radiation hybrid mapping.* Genomics, 2002. **79**(3): p. 451-7.
73. Fu, X.-F., et al., *DAZ Family Proteins, Key Players for Germ Cell Development.* International Journal of Biological Sciences, 2015. **11**: p. 1226 - 1235.
74. Hayashi, M., et al., *Generation of germ cells from pluripotent stem cells in mammals.* Reproductive Medicine and Biology, 2018. **17**(2): p. 107-114.

75. Hopkins, J., et al., *Meiosis-Specific Cohesin Component, Stag3 Is Essential for Maintaining Centromere Chromatid Cohesion, and Required for DNA Repair and Synapsis between Homologous Chromosomes.* PLOS Genetics, 2014. **10**(7): p. e1004413.
76. Schwickart, M., et al., *Swm1/Apc13 Is an Evolutionarily Conserved Subunit of the Anaphase-Promoting Complex Stabilizing the Association of Cdc16 and Cdc27.* Molecular and Cellular Biology, 2004. **24**(8): p. 3562-3576.
77. Yin, F.-X., et al., *SGO1 Maintains Bovine Meiotic and Mitotic Centromeric Cohesions of Sister Chromatids and Directly Affects Embryo Development.* PLOS ONE, 2013. **8**(9): p. e73636.
78. Leem, J., J.-S. Kim, and J.S. Oh, *Oocytes can repair DNA damage during meiosis via a microtubule-dependent recruitment of CIP2A–MDC1–TOPBP1 complex from spindle pole to chromosomes.* Nucleic Acids Research, 2023. **51**(10): p. 4899-4913.
79. Gilad, O., et al., *Combining ATR Suppression with Oncogenic Ras Synergistically Increases Genomic Instability, Causing Synthetic Lethality or Tumorigenesis in a Dosage-Dependent Manner.* Cancer Research, 2010. **70**(23): p. 9693-9702.
80. Ma, G., et al., *Roles of ZIC family genes in human gastric cancer.* Int J Mol Med, 2016. **38**(1): p. 259-266.
